# Advances in immune-inflammatory interactions within the testicular-penile microenvironment of varicocele patients: molecular mechanisms underlying erectile dysfunction and spermatogenic impairment

**DOI:** 10.3389/fendo.2026.1813721

**Published:** 2026-05-25

**Authors:** Gaoyuan Xu, Qingfeng Yu

**Affiliations:** 1 Department of Reproductive Medicine, The 960th Hospital of the PLA Joint Logistics Support Force, Jinan, China; 2 First Department of Cadre Ward, The 960th Hospital of the PLA Joint Logistics Support Force, Jinan, China

**Keywords:** erectile dysfunction, immune-inflammatory interaction, molecular mechanisms, penile microenvironment, spermatogenic impairment, testicular microenvironment, varicocele

## Abstract

Varicocele is a prevalent cause of male infertility and has been associated with erectile dysfunction in clinical and preclinical studies, characterized by a complex pathophysiological process involving immune-inflammatory responses. This review focuses on the preclinically supported immune-inflammatory interactions between the testicular and penile microenvironments in varicocele patients, systematically summarizing the plausible and partially validated molecular mechanisms mediating erectile dysfunction and spermatogenic impairment. We explore the network relationships among local and systemic inflammatory cytokines, immune cell infiltration, oxidative stress, endothelial dysfunction, and neurovascular injury. By integrating recent omics studies, animal models, and available clinical data, this article elucidates the hypothetical and mechanistically plausible pathological linkage from abnormal varicose veins in the pampiniform plexus to distal impairment of penile cavernous function. The review aims to synthesize and contextualize the systemic impact of varicocele and to establish a theoretical foundation for developing targeted therapies addressing immune-inflammatory pathways.

## 1 Introduction

Varicocele, a common vascular abnormality in men, has been increasingly recognized not just as a localized issue affecting venous return from the testis but as a condition that may have systemic implications on male reproductive health (1, 2). The pathophysiological mechanisms underlying varicocele are complex and involve a multifaceted interplay of immune and inflammatory responses that can extend beyond the testicular microenvironment to potentially impact erectile function and fertility (3–6). Recent studies, including both clinical observations and experimental investigations, have suggested that varicocele is associated with a chronic inflammatory state characterized by elevated pro-inflammatory cytokines, oxidative stress, and disruption of the blood-testis barrier. These alterations are well supported in relation to spermatogenic dysfunction, whereas their contribution to erectile dysfunction (ED) remains less clearly established in human studies (3, 4, 7).

In the testicular microenvironment, venous congestion associated with varicocele leads to increased temperature, hypoxia, and accumulation of metabolic waste products (7–11). These factors have been shown, particularly in experimental models and supported by limited human data, to trigger a cascade of immune responses, including activation of macrophages and increased production of reactive oxygen species (ROS) and inflammatory cytokines such as TNF-α, IL-1β, and IL-6 (3, 12). The resultant chronic inflammatory state not only damages spermatogenic cells but also affects the function of Sertoli and Leydig cells, which are crucial for spermatogenesis and testosterone production, respectively (13). Furthermore, preclinical evidence, primarily derived from animal models, suggests that inflammatory mediators released into the systemic circulation may disseminate to distant organs, including the penis, thereby providing a mechanistic hypothesis linking testicular pathology and erectile dysfunction (14, 15). However, direct clinical evidence supporting this systemic testis–penis interaction in humans remains limited.

Clinical evidence supporting a direct and robust causal relationship between varicocele and erectile dysfunction remains limited and inconsistent, with most available data derived from observational studies that show associations rather than causality Nonetheless, preclinical and mechanistic studies indicate that varicocele-induced oxidative stress and inflammatory cytokines can compromise endothelial function in the penile vasculature (16, 17). Although this mechanism has not been conclusively demonstrated in human clinical studies. This impairment is characterized by reduced nitric oxide (NO) bioavailability, which is essential for achieving and maintaining an erection (18). Studies, predominantly in animal models, have indicated that varicocele-induced oxidative stress can lead to endothelial dysfunction in the corpus cavernosum, resulting in impaired relaxation responses and increased collagen deposition, which ultimately contributes to veno-occlusive dysfunction (12, 19).

Moreover, preclinical investigations have highlighted the interplay between inflammation and the neurovascular components of the penis, suggesting that the inflammatory milieu can disrupt nerve function and induce apoptosis of cavernous nerve fibers in experimental settings (14, 20, 21). Such findings, however, are largely limited to animal studies and have not yet been validated in human populations. The activation of the NLRP3 inflammasome has been implicated in this process, linking chronic inflammation to both testicular dysfunction and erectile impairment (13, 14). In this context, the hypothesized immune-inflammatory interactions between the testicular and penile microenvironments emerge as biologically plausible pathways, primarily supported by preclinical evidence, that may connect varicocele to male infertility and potentially contribute to sexual dysfunction (22).

In summary, the pathophysiology of varicocele extends beyond local testicular effects, encompassing a broader immune-inflammatory network that clearly influences spermatogenesis and may also influence erectile function, although the latter is supported predominantly by indirect and preclinical evidence. The chronic inflammatory state induced by varicocele, characterized by elevated cytokines and oxidative stress, plays a pivotal role in testicular dysfunction and represents a plausible, yet not definitively established, contributor to erectile dysfunction. To improve conceptual clarity, this review explicitly distinguishes between evidence derived from human clinical studies, animal models, *in vitro* experiments, and extrapolation from broader erectile dysfunction research. Understanding these interconnections is essential for developing targeted therapeutic strategies aimed at mitigating the adverse effects of varicocele on male reproductive health. The exploration of molecular mechanisms and signaling pathways involved in these interactions will be crucial for future research and clinical applications aimed at improving outcomes for men suffering from varicocele-related infertility and potential erectile dysfunction.

## 2 Immune inflammatory activation of testicular microenvironment induced by varicocele

**Figure 1:**
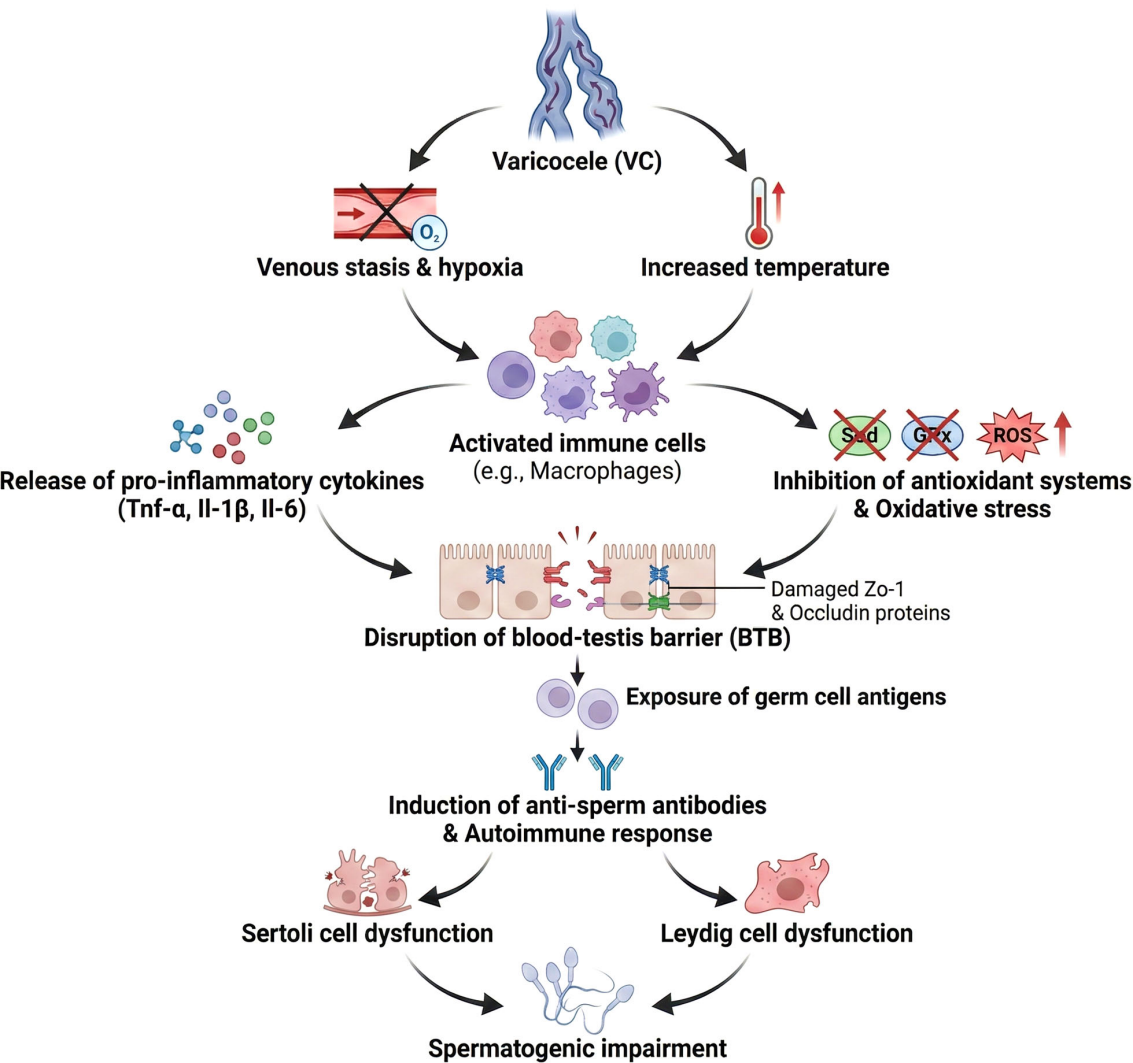
Schematic diagram of the immune-inflammatory cascade and spermatogenic impairment in the testicular microenvironment induced by varicocele. Varicocele leads to venous stasis, hypoxia, and increased testicular temperature in the testicular microenvironment, which activates immune cells (e.g., macrophages) in the testicular interstitium. These activated immune cells secrete pro-inflammatory cytokines (TNF-α, IL-1β, IL-6) and induce overproduction of ROS via mitochondrial dysfunction and NADPH oxidase activation. On one hand, excessive ROS breaks the redox balance by suppressing the antioxidant defense system (e.g., SOD, GPx), causing oxidative damage to spermatogenic cells; on the other hand, pro-inflammatory cytokines and ROS disrupt the integrity of the blood-testis barrier (BTB) by damaging tight junction proteins (ZO-1, occludin), exposing germ cell antigens to the immune system and inducing the production of anti-sperm antibodies. Collectively, these pathological processes impair the function of Sertoli and Leydig cells, ultimately leading to spermatogenic impairment.

Varicocele represents one of the most prevalent causes of male infertility worldwide, with its pathological manifestations closely tied to the disruption of testicular microenvironmental homeostasis (23, 24). Accumulating clinical and basic research evidence has identified immune inflammatory activation as a central and initiating driver of varicocele-induced testicular dysfunction, which triggers a cascade of pathological events including abnormal inflammatory factor secretion, redox balance disorder and structural damage of the testicular protective barrier, ultimately impairing spermatogenic function and male reproductive health (25). This section will systematically elaborate on the multi-dimensional mechanisms underlying immune inflammatory activation in the testicular microenvironment induced by varicocele, as well as the sequential pathological consequences and their regulatory networks, aiming to clarify the core role of immune inflammatory dysregulation in the pathogenesis of varicocele-related male infertility and lay a theoretical foundation for exploring targeted anti-inflammatory and microenvironment-regulating therapeutic strategies for this condition (**Figure 1**) (12).

### 2.1 Activation and regulation of local inflammatory factor networks

The pathophysiology of varicocele is closely linked to local inflammatory responses within the testicular microenvironment, particularly due to the effects of venous stasis (9, 13, 26, 27). The accumulation of blood in the pampiniform plexus leads to increased testicular temperature and hypoxia, creating a milieu conducive to the activation of immune cells such as macrophages and mast cells in the testicular interstitium (3, 12, 13, 26). These cells are pivotal in the inflammatory response, as they release a variety of pro-inflammatory cytokines, including TNF-α, IL-1β, and IL-6 (13). The release of these cytokines is not merely a passive response; rather, it initiates a cascade of signaling events that amplify the inflammatory response through autocrine and paracrine mechanisms (28, 29). This process activates critical inflammatory signaling pathways, including nuclear factor kappa B (NF-κB) and mitogen-activated protein kinase (MAPK), which further perpetuate the inflammatory cycle by enhancing the production of additional cytokines and inflammatory mediators (29). The positive feedback loop established by these interactions not only exacerbates local inflammation but also contributes to the dysfunction of Sertoli and Leydig cells, ultimately leading to impaired spermatogenesis and erectile dysfunction (3, 27). Recent studies have also highlighted the role of an imbalance in anti-inflammatory factors, such as IL-10 and TGF-β, which fail to counteract the persistent inflammatory state induced by the overexpression of pro-inflammatory cytokines (29–31). This dysregulation is further influenced by specific microRNAs, such as miR-155 and miR-146a, which have been shown to play crucial roles in modulating the balance between pro-inflammatory and anti-inflammatory responses within the testicular environment (32, 33). These microRNAs can either promote or inhibit the expression of various cytokines, thus serving as key regulators in the inflammatory network that characterizes varicocele pathology (34). Understanding these intricate interactions and the molecular mechanisms underlying them is essential for developing targeted therapeutic strategies aimed at mitigating the inflammatory processes associated with varicocele and its related complications (3). The interplay between local inflammatory factors and the testicular microenvironment underscores the complexity of varicocele as a condition that not only affects fertility but also has broader implications for male reproductive health (3, 25).

### 2.2 The collapse of the antioxidant defense system and oxidative stress

The interplay between oxidative stress and the collapse of the antioxidant defense system is a critical factor in the pathophysiology of conditions such as varicocele, which is a significant contributor to male infertility (35). In patients with varicocele, hypoxia and inflammatory cell infiltration lead to mitochondrial dysfunction and the activation of NADPH oxidase, resulting in the overproduction of ROS, including superoxide anions and hydrogen peroxide (36). These ROS can cause extensive oxidative damage to sperm cells by attacking membrane lipids, proteins, and DNA, leading to lipid peroxidation, loss of protein function, and DNA fragmentation (37–39). This oxidative damage directly impairs spermatogenesis, contributing to infertility (39). Concurrently, the endogenous antioxidant systems within the testes, such as superoxide dismutase (SOD) and glutathione peroxidase (GPx), are often found to be suppressed in varicocele patients (40, 41). This suppression hampers the testes’ ability to effectively neutralize excess ROS, creating a vicious cycle of oxidative stress that exacerbates cellular damage and further compromises sperm quality (11, 42). The resulting oxidative stress not only affects sperm production but also has implications for overall reproductive health, highlighting the importance of maintaining a balanced redox environment within the male reproductive system to preserve fertility and prevent long-term consequences associated with oxidative damage (35, 43, 44).

In the context of varicocele, the mechanisms of oxidative stress are multifaceted. The excessive ROS production can overwhelm the antioxidant defenses, leading to a state of oxidative stress that is detrimental to testicular function (35, 45, 46). This imbalance can initiate a cascade of cellular events that ultimately result in apoptosis of germ cells and impaired spermatogenesis (43, 47). Moreover, the detrimental effects of oxidative stress are not limited to sperm cells alone; they can also affect the surrounding somatic cells within the testes, further compromising the microenvironment necessary for optimal spermatogenesis (4, 48). The interplay between oxidative stress and the collapse of the antioxidant defense system underscores the need for therapeutic strategies aimed at restoring redox balance, such as the use of antioxidants, to mitigate the effects of oxidative damage and improve fertility outcomes in men suffering from varicocele-related infertility (4, 46, 48–50).

Experimental varicocele models provide additional mechanistic support for this redox imbalance (4). In rodent models of varicocele, testicular tissue frequently exhibits degeneration of the spermatogenic epithelium, germ-cell apoptosis, mitochondrial injury, inflammatory-cell infiltration, and heightened oxidative stress (43, 51). These pathological changes indicate that oxidative stress is not merely a biochemical abnormality but a direct driver of structural and functional deterioration in the seminiferous epithelium (4, 27). In this context, ROS accumulation may amplify inflammatory signaling by activating redox-sensitive pathways such as NF-κB, MAPK, and inflammasome-related cascades, thereby linking oxidative stress to cytokine production, immune-cell recruitment, and progressive testicular microenvironmental damage (13). Conversely, inflammatory cytokines can further promote mitochondrial dysfunction and ROS generation, forming a self-perpetuating cycle between oxidative stress and inflammation (43).

Recent research has also explored the potential of various antioxidant therapies to alleviate oxidative stress in varicocele patients (52). For instance, clinical trials have indicated that interventions aimed at reducing oxidative stress, such as the administration of oral antioxidants, may improve semen parameters and enhance fertility outcomes in men with varicocele (52). However, the evidence remains heterogeneous, and further studies are necessary to establish standardized treatment protocols and assess the long-term benefits of such interventions (53–55). The complexity of oxidative stress and its impact on male fertility necessitates a comprehensive understanding of the underlying molecular mechanisms, which could lead to the development of targeted therapies that not only address the symptoms of infertility but also tackle the root causes related to oxidative damage and antioxidant system collapse (47, 55–58).

In conclusion, the collapse of the antioxidant defense system in the face of oxidative stress represents a critical pathway in the etiology of infertility associated with varicocele. Understanding the mechanisms by which oxidative stress affects spermatogenesis and the role of antioxidant defenses is essential for developing effective therapeutic strategies aimed at improving male reproductive health. Future research should focus on elucidating the precise molecular pathways involved in oxidative stress and exploring novel antioxidant therapies that could restore balance to the redox environment within the testes, ultimately enhancing fertility outcomes in affected individuals (59–61).

### 2.3 Destruction of the blood-testis barrier and induction of autoimmune responses

The blood-testis barrier (BTB) serves as a critical protective structure that maintains the immunological privilege of the testicular environment, allowing for the development of germ cells without interference from the immune system (62–64). However, persistent inflammation and oxidative stress can compromise the integrity of this barrier, particularly affecting the tight junction proteins such as ZO-1 and occludin, which are essential for maintaining the structural and functional integrity of the BTB (65). When these proteins are disrupted, the barrier becomes permeable, exposing normally sequestered germ cell antigens to the immune system. This exposure can lead to the generation of anti-sperm antibodies, triggering an autoimmune response that targets the spermatozoa and other germ cells (66). The presence of these autoantibodies can further exacerbate local inflammation, creating a vicious cycle that not only impairs spermatogenesis but may also have systemic effects, potentially leading to broader reproductive dysfunction (67).

Moreover, the breakdown of the BTB is not merely a localized phenomenon; it can have far-reaching implications for male fertility. Once the immune system recognizes the exposed germ cell antigens as foreign, it may mount a systemic immune response that could affect other organs, including the penis, thereby contributing to erectile dysfunction (68). The inflammatory cytokines released during this process, such as TNF-α and IL-6, can further disrupt the delicate balance of the testicular microenvironment, leading to additional damage to the BTB and a decline in testosterone production, which is vital for male reproductive health (69).

In summary, the destruction of the blood-testis barrier due to chronic inflammation and oxidative stress not only facilitates the exposure of germ cell antigens to the immune system but also initiates a cascade of autoimmune responses that can significantly impair male reproductive function. This complex interplay between barrier integrity, immune response, and reproductive health underscores the importance of maintaining the BTB and managing inflammation to preserve male fertility (68).

## 3 Release of systemic inflammatory factors and effects on distant organs

**Figure 2:**
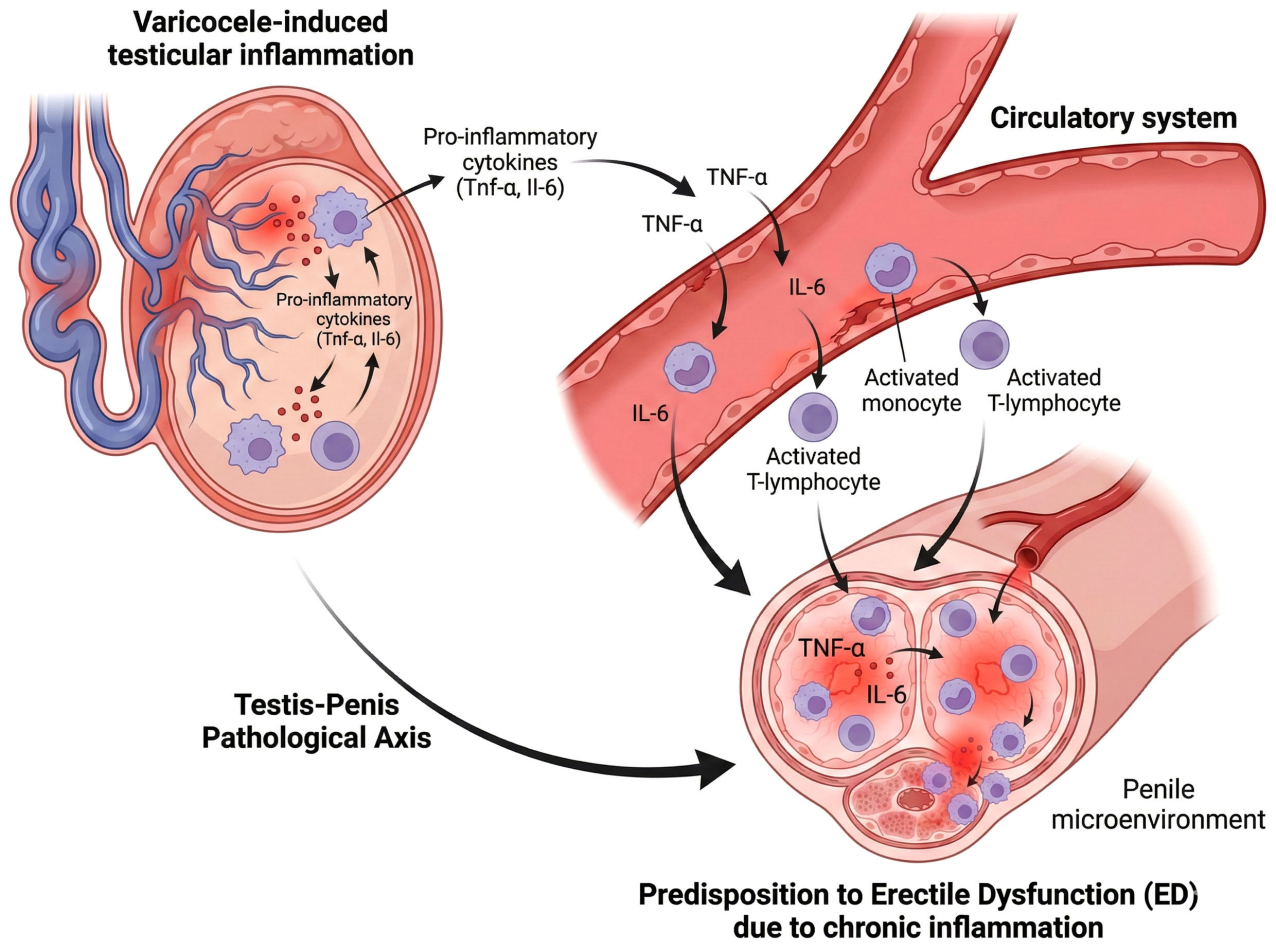
Schematic diagram of systemic inflammatory dissemination induced by varicocele and its distant effects on the penile microenvironment. Varicocele-induced chronic inflammation in the testicular microenvironment leads to two key pathological processes: (1) The local overproduced pro-inflammatory cytokines (TNF-α, IL-6) spill into the systemic circulation through the damaged testicular vascular endothelium, forming a state of low-grade systemic inflammation; (2) The inflammatory microenvironment activates testicular interstitial immune cells (e.g., macrophages, T lymphocytes), which upregulate adhesion molecule expression and migrate to distant organs via the circulation. These “pathological messengers” (circulating inflammatory cytokines and activated immune cells) target the penile microenvironment, inducing subsequent structural and functional abnormalities (e.g., endothelial dysfunction, smooth muscle cell damage, nerve injury) and ultimately contributing to erectile dysfunction. Notably, animal studies have confirmed that immune cell infiltration is increased in both the testis and penile corpus cavernosum of varicocele models, verifying the systemic nature of this inflammatory response.

Varicocele, as a leading cause of male infertility, is increasingly recognized to induce not only localized testicular inflammation but also a cascade of systemic inflammatory responses that extend beyond the reproductive tract (1, 4). Mounting evidence, derived from both clinical observations and experimental studies, suggests that disruption of testicular homeostasis and vascular integrity in varicocele may lead to the dissemination of inflammatory mediators and activation of immune cells in the systemic circulation (3, 15, 29, 70). However, the extent to which these systemic alterations exert direct effects on distant organs, particularly the penis, remains incompletely defined in human studies. This section will systematically elaborate on two key processes underlying this proposed systemic inflammatory cascade—namely, the overflow of testicular inflammatory mediators into the circulation and the activation and migration of immune cells—and discuss their potential implications for distant organ function (**Figure 2**).

### 3.1 Overflow of inflammatory mediators from the testis into the circulatory system

The testis is a complex organ that not only plays a crucial role in spermatogenesis but also serves as an immunologically privileged site, where local inflammatory responses can significantly influence systemic health (71, 72). In the context of varicocele, several clinical studies have reported elevated levels of pro-inflammatory cytokines such as IL-6 and TNF-α in the systemic circulation (3, 15, 73). These findings suggest, but do not directly prove, that local testicular inflammation may contribute to systemic inflammatory changes. The mechanism by which these cytokines enter the systemic circulation is not fully elucidated in humans, but endothelial dysfunction and altered vascular permeability have been proposed based largely on experimental studies (3). Clinical studies have demonstrated that serum levels of IL-6, TNF-α, and C-reactive protein (CRP) are markedly elevated in patients with varicocele compared to healthy controls, providing compelling evidence for the presence of this systemic inflammatory response (3, 73). Such circulating inflammatory factors may act as systemic mediators; however, their direct effects on distal organs, including the penis, have not been conclusively demonstrated in human studies (74, 75). Evidence supporting penile endothelial dysfunction secondary to systemic inflammation is largely extrapolated from general ED research and preclinical models. This is particularly relevant given the established association between systemic inflammation and erectile dysfunction in the broader literature, although its specific contribution in the context of varicocele remains to be clarified (3, 4, 74). Furthermore, the potential interplay between local testicular inflammation and systemic effects underscores the importance of considering varicocele as a condition with possible systemic implications, although definitive causal pathways remain to be established (4, 74, 76). The findings suggest that therapeutic strategies aimed at mitigating inflammation in the testis could have far-reaching implications for improving both fertility outcomes and the overall health of affected individuals (77, 78). The intricate relationship between local and systemic inflammation highlights the need for a comprehensive approach to understanding and treating conditions like varicocele, where the testicular microenvironment plays a pivotal role in male reproductive health (75).

### 3.2 Activation and migration of immune cells

The inflammatory microenvironment within the testes may activate circulating monocytes, macrophages, and T lymphocytes, thereby facilitating their recruitment to sites of tissue injury or inflammatory stimulation (79, 80). In varicocele, venous stasis, hypoxia, hyperthermia, oxidative stress, and local cytokine accumulation collectively create a pro-inflammatory milieu that favors immune-cell activation within the testicular interstitium (81). Mechanistically, inflammatory cytokines and chemokines may increase the expression of adhesion molecules and chemokine receptors on circulating immune cells, thereby promoting their adhesion to vascular endothelium and subsequent migration into inflamed tissues (82). This phenomenon has been observed in systemic inflammatory conditions; however, its occurrence in varicocele patients has not been directly demonstrated (83). For instance, the recruitment of these activated immune cells to the penis can exacerbate local inflammation, potentially contributing to conditions such as erectile dysfunction and spermatogenic impairment (84–86). The interplay between the immune system and the local microenvironment is crucial for understanding how systemic inflammation can influence localized tissue responses and pathology (87, 88).

Experimental varicocele models provide important preclinical support for this hypothesis. In rodent models of varicocele, histological changes include degeneration of the spermatogenic epithelium, disruption of seminiferous tubule architecture, inflammatory-cell infiltration, and increased oxidative stress within the testicular tissue (51, 89, 90). These pathological phenotypes suggest that immune activation is not merely a secondary consequence of tissue injury but may participate actively in the progression of varicocele-induced testicular dysfunction (3). In particular, increased infiltration of macrophages and T cells has been observed in the testes after experimental varicocele induction, supporting the concept that local inflammatory signaling can amplify immune-cell recruitment and sustain a chronic inflammatory state (15, 91). Importantly, some experimental studies have further reported immune-cell infiltration not only in the testes but also in the penile corpus cavernosum after varicocele induction (51). This dual-site infiltration provides preclinical evidence for a potential systemic immune-inflammatory response linking the testicular and penile microenvironments (27). In these models, the coexistence of testicular inflammatory injury and inflammatory changes in erectile tissue suggests that shared circulating mediators, including cytokines, chemokines, and oxidative stress-related signals, may contribute to coordinated tissue responses in both organs. However, such dual-site immune-cell infiltration has not yet been confirmed in human varicocele cohorts. Therefore, these animal-model findings should be interpreted as mechanistic support for the proposed testis–penis inflammatory axis rather than as direct clinical evidence of causality.

The causal involvement of TNF-α signaling in this inflammatory cascade has been further supported by gene-knockout evidence (3, 12). In experimental varicocele models using TNF-α receptor knockout mice, pathological changes in both the testes and the penis were significantly attenuated compared with wild-type controls (92–94). This finding suggests that TNF-α is not merely a downstream inflammatory marker but may actively drive varicocele-induced immune-inflammatory injury and dual-organ dysfunction (3, 15, 95). Mechanistically, TNF-α receptor signaling may promote immune-cell recruitment, cytokine amplification, oxidative stress, and activation of downstream inflammatory pathways such as NF-κB and MAPK (4, 96). Therefore, TNF-α receptor knockout findings provide pathway-specific preclinical evidence supporting the central role of TNF-α in immune-cell activation and migration within the proposed testicular–penile inflammatory axis (97). Nevertheless, because these results are derived from experimental models, their relevance to human varicocele-associated erectile dysfunction and spermatogenic impairment requires further clinical validation (98, 99).

A theoretical hypothesis proposes that “pre-activated” immune cells may preferentially home to tissues vulnerable to microcirculatory disturbances, such as erectile tissue (84, 100, 101). This concept is based primarily on extrapolation from vascular inflammation studies rather than direct evidence in varicocele. This phenomenon is particularly concerning as it may lead to a vicious cycle of inflammation (101). Once these immune cells infiltrate the erectile tissue, they can release pro-inflammatory cytokines and chemokines that further recruit additional immune cells, thereby amplifying the inflammatory response (84). This process can disrupt normal vascular function, leading to impaired blood flow and contributing to erectile dysfunction. The chronic presence of these activated immune cells may also result in tissue remodeling and fibrosis, further complicating the clinical picture. Understanding the mechanisms behind this aberrant homing and the subsequent inflammatory cascade is essential for developing therapeutic strategies aimed at mitigating the effects of chronic inflammation in the penile tissue (102).

Animal model studies have demonstrated that induction of varicocele is associated with increased immune cell infiltration in both the testis and the corpus cavernosum (51). Importantly, such dual-site immune cell infiltration has not been confirmed in human studies and should therefore be interpreted as preclinical evidence. This infiltration, characterized by increased macrophages and T cells, suggests a systemic inflammatory response in experimental settings (51). These findings suggest a coordinated immune response potentially driven by shared inflammatory mediators, although this remains to be validated clinically (51). This dual infiltration highlights the interconnectedness of the reproductive and erectile functions and underscores the importance of considering both sites when evaluating the impact of varicocele on male reproductive health (51, 98, 103). The findings from these studies suggest that targeting the inflammatory pathways involved in immune cell activation and migration could be a potential therapeutic avenue for addressing both spermatogenic dysfunction and erectile impairment associated with varicocele (104).

In summary, the activation and migration of immune cells in response to inflammatory stimuli within the testicular microenvironment play a critical role in the pathogenesis of conditions such as erectile dysfunction and spermatogenic impairment. The ability of these cells to migrate to tissues at risk of inflammation exacerbates local inflammatory responses, creating a cycle that can lead to further tissue damage and dysfunction. Understanding these mechanisms is vital for developing targeted interventions that could alleviate the adverse effects of chronic inflammation in male reproductive health.

## 4 Pathophysiological mechanisms linking testicular and penile microenvironments

**Figure 3:**
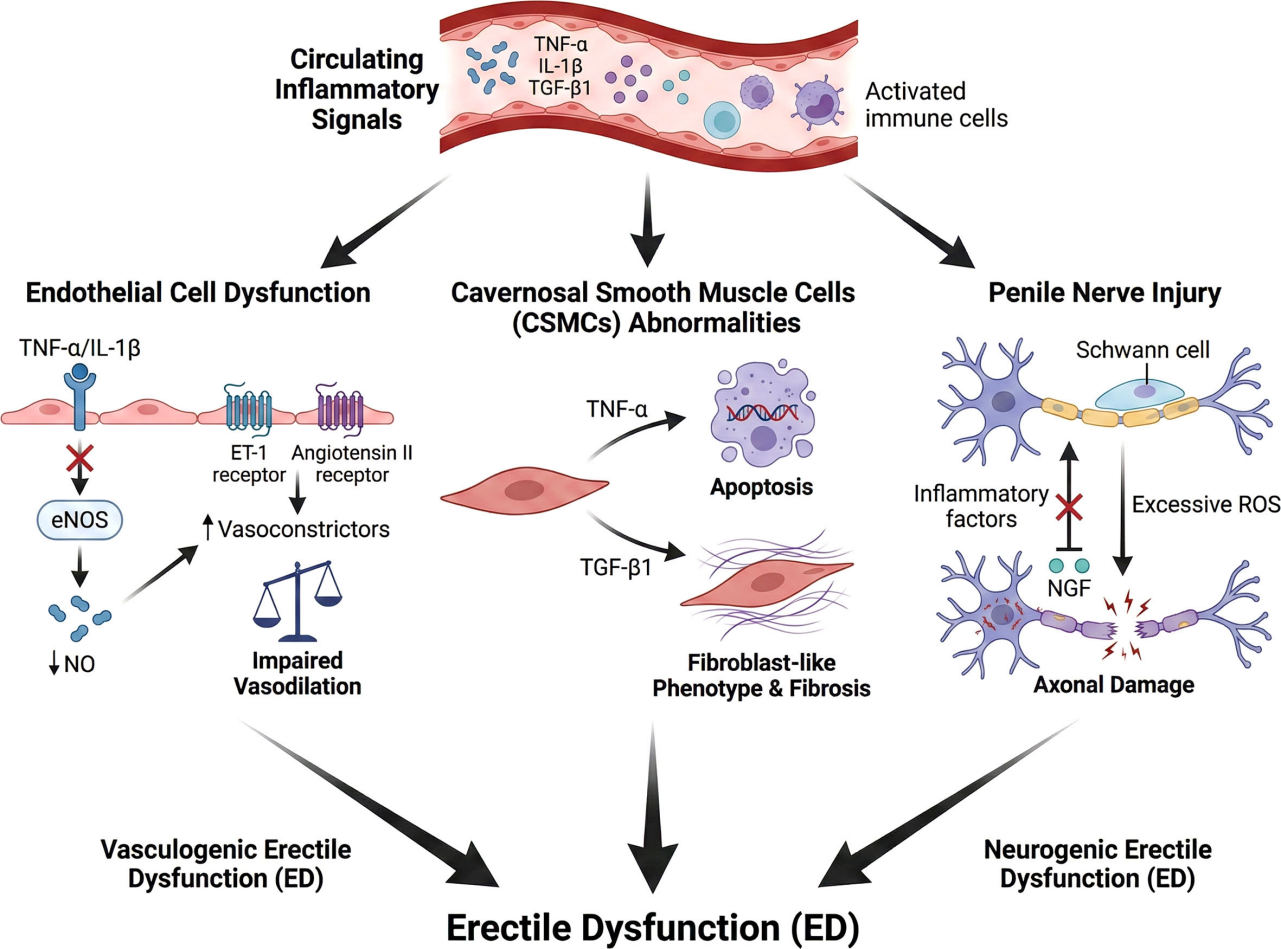
Schematic diagram of the penile microenvironment’s response to systemic inflammatory signals and local inflammatory amplification in varicocele patients. Systemic inflammatory signals (circulating pro-inflammatory cytokines such as TNF-α, IL-1β, and activated immune cells) derived from varicocele-induced testicular inflammation target the penile microenvironment, triggering three key pathological processes: (1) Endothelial cell dysfunction: Inflammatory cytokines inhibit the activity and expression of endothelial nitric oxide synthase (eNOS), reduce nitric oxide (NO) bioavailability, and promote the release of vasoconstrictors such as endothelin-1 (ET-1), disrupting the balance between vasodilation and vasoconstriction; (2) Phenotypic transformation and apoptosis of cavernous smooth muscle cells (CSMCs): TNF-α activates the caspase pathway to induce CSMC apoptosis, while TGF-β1 promotes their transformation into fibroblast-like cells, leading to cavernous fibrosis and reduced tissue distensibility; (3) Inflammatory damage to penile nerves: Inflammatory cytokines inhibit the expression of nerve growth factor (NGF) and induce excessive production of neurotoxic NO via iNOS, impairing cavernous nerve integrity and neurotransmitter release. These local pathological changes are further amplified by the inflammatory cascade within the penile tissue, ultimately resulting in erectile dysfunction.

The penile microenvironment, composed of endothelial cells, cavernous smooth muscle cells, penile nerves, and the extracellular matrix, maintains the structural and functional integrity essential for normal erectile function. Systemic inflammatory signals, originating from various pathological conditions such as metabolic disorders, chronic infections, or autoimmune diseases, have been shown to affect penile tissue and trigger local inflammatory amplification in the broader ED literature. However, it should be emphasized that the majority of mechanisms described below are well-established in general ED research, whereas their specific involvement in varicocele-associated ED is supported predominantly by indirect evidence and preclinical studies rather than direct human clinical data. This local inflammatory response not only directly impairs the physiological functions of key cellular components in the penis but also forms a vicious cycle with oxidative stress and tissue remodeling, ultimately contributing to the development and progression of ED (**Figure 3**).

### 4.1 Endothelial dysfunction and vasoconstrictive imbalance

Endothelial dysfunction represents a central pathological outcome through which systemic inflammatory signals may impair penile vascular homeostasis and contribute to ED (105–107). In the penile corpus cavernosum, endothelial cells regulate vascular relaxation mainly through the endothelial nitric oxide synthase (eNOS)/nitric oxide (NO) pathway (108, 109). NO is a crucial vasodilatory mediator that promotes cavernous smooth muscle relaxation, increases arterial inflow, and facilitates sinusoidal expansion during erection (110, 111). Therefore, any sustained reduction in eNOS activity or NO bioavailability can directly disturb the hemodynamic basis of erectile function.

In varicocele, circulating inflammatory mediators such as TNF-α, IL-1β, and IL-6 may act on endothelial cells and suppress eNOS expression or activity (27, 106, 112). Inflammatory cytokines may also promote ROS generation, which further reduces NO bioavailability by accelerating NO degradation and increasing oxidative stress (61, 106). As a result, the penile vascular environment shifts from a vasodilatory state toward impaired relaxation, increased vascular resistance, and insufficient cavernous perfusion (61, 98, 113). Given the relatively small caliber of the penile resistance vasculature, even mild endothelial injury or reduced vascular compliance may become clinically relevant earlier in the penis than in larger vascular beds (114, 115).

This endothelial imbalance is further amplified by vasoconstrictive signaling (116, 117). Inflammatory cytokines can stimulate endothelin-1 (ET-1) release and activate the angiotensin II pathway, thereby promoting vascular smooth muscle contraction and reducing penile arterial inflow (118, 119). In parallel, the RhoA/ROCK pathway provides an additional molecular explanation for impaired endothelial relaxation. ROCK activation can suppress eNOS activity and reduce NO-mediated vasodilation, while simultaneously favoring smooth muscle contraction (111, 120). Therefore, rather than discussing RhoA/ROCK as a separate pathway, it is more coherent to consider it a key amplifier of endothelial dysfunction and vasoconstrictive imbalance in the penile microenvironment.

Experimental varicocele models provide preclinical support for this endothelial mechanism. In these models, varicocele induction has been associated with increased inflammatory cytokine expression, decreased eNOS activity, and impaired endothelial function in penile tissue (27, 108). Further evidence from eNOS knockout models reinforces the importance of NO signaling in maintaining reproductive vascular homeostasis (120, 121). When eNOS knockout mice are subjected to experimental varicocele induction, impaired NO production appears to aggravate vascular dysfunction and testicular injury, suggesting that eNOS-derived NO is essential for preserving local blood flow, endothelial integrity, and tissue homeostasis under varicocele-related stress conditions (122, 123). These findings support the concept that defective eNOS/NO signaling may serve as a key molecular bridge between inflammatory vascular injury and reproductive dysfunction. Nevertheless, because these data are derived from genetically modified experimental systems, their applicability to human varicocele-associated ED requires further validation through clinical endothelial-function assessment and penile hemodynamic studies.

The AGE–RAGE axis may also contribute to endothelial injury through oxidative and inflammatory amplification (4). Varicocele-induced oxidative stress may promote the formation of advanced glycation end products (AGEs), which bind to the receptor for AGEs (RAGE) and activate downstream NF-κB signaling (4, 124). This process enhances cytokine production, oxidative stress, and endothelial inflammation, all of which may further impair NO signaling and vascular relaxation (125, 126). RAGE knockout models provide mechanistic support for the receptor-level contribution of RAGE signaling to inflammatory tissue injury, although direct evidence in human varicocele-associated ED remains limited (12). Therefore, AGE–RAGE signaling should be interpreted as a plausible endothelial-amplifying pathway rather than as a clinically proven mechanism in varicocele-related penile dysfunction.

Sympathetic nervous system overactivity and catecholamine release may further aggravate endothelial and vascular dysfunction (127). Elevated norepinephrine can induce vasoconstriction through α-adrenergic receptor activation in both testicular and penile vasculature, thereby compromising testicular perfusion and opposing the smooth muscle relaxation required for erection (128, 129). In this context, sympathetic overactivity should not be viewed as an isolated mechanism but as part of a broader vasoconstrictive network that converges with inflammatory cytokines, RhoA/ROCK activation, ET-1, angiotensin II, and reduced NO bioavailability (130).

In summary, endothelial dysfunction in varicocele-associated ED may result from the convergence of inflammatory cytokines, oxidative stress, eNOS/NO suppression, RhoA/ROCK activation, AGE–RAGE/NF-κB signaling, and sympathetic catecholamine-mediated vasoconstriction. These mechanisms collectively shift the penile microenvironment toward impaired vasodilation, increased vascular resistance, and reduced cavernous perfusion. Although experimental evidence supports this pathway, direct clinical confirmation in patients with varicocele remains insufficient.

### 4.2 Cavernous smooth muscle abnormalities, contractile imbalance, and fibrosis

Cavernous smooth muscle cells (CSMCs) are essential for the structural and functional integrity of the corpus cavernosum (131). During erection, relaxation of CSMCs permits sinusoidal expansion, increased arterial inflow, and effective veno-occlusion (131, 132). Therefore, CSMC apoptosis, phenotypic transformation, excessive contractility, or extracellular matrix remodeling can reduce cavernous compliance and contribute to organic ED (111, 133). Inflammatory mediators, particularly TNF-α, may directly impair CSMC survival (134). TNF-α has been shown, mainly in experimental ED models and chronic inflammatory or metabolic conditions, to activate caspase-dependent apoptotic pathways while inhibiting the PI3K/Akt survival pathway (111, 134). This dual effect promotes CSMC apoptosis and reduces the number of functional smooth muscle cells necessary for adequate penile rigidity (134). In the context of varicocele, systemic inflammatory dissemination from the testicular microenvironment may therefore contribute to CSMC loss, although direct human evidence remains limited (25).

In addition to apoptosis, inflammatory and oxidative stress signals may promote CSMC phenotypic transformation (135). Transforming growth factor-beta 1 (TGF-β1) is a key profibrotic mediator that drives the transition of CSMCs from a contractile phenotype toward a synthetic or fibroblast-like phenotype (136). This transition is characterized by increased extracellular matrix production, collagen deposition, and progressive remodeling of the corpus cavernosum (135, 137, 138). As smooth muscle content decreases and collagen content increases, the smooth muscle-to-collagen ratio declines, reducing cavernous elasticity and impairing the veno-occlusive mechanism required for erection (138–140).

Experimental varicocele models have reported fibrotic changes within penile tissue, suggesting that varicocele-induced inflammatory and oxidative stress signals may extend beyond endothelial dysfunction and contribute to structural remodeling of the corpus cavernosum (51). These findings provide preclinical support for the hypothesis that the testicular inflammatory microenvironment may influence penile smooth muscle integrity and extracellular matrix homeostasis (3). However, direct histological evidence of cavernous fibrosis in patients with varicocele-associated ED is still lacking (12, 141). RhoA/ROCK activation is particularly relevant to this pathological outcome because it directly enhances CSMC contraction and contributes to contractile imbalance (130, 131). In penile tissue, ROCK activation promotes sustained smooth muscle contraction, counteracting the relaxation required for erection (142, 143). At the same time, ROCK-mediated suppression of eNOS/NO signaling further reduces the vasodilatory input necessary for smooth muscle relaxation (120). Thus, RhoA/ROCK should be integrated into the discussion of CSMC dysfunction rather than presented as a separate molecular pathway (131). Its activation links inflammation, vasoconstriction, reduced NO signaling, and smooth muscle hypercontractility within a single pathological cascade. AGE–RAGE signaling may also promote CSMC remodeling indirectly by amplifying oxidative stress and NF-κB-mediated inflammation (144). Persistent AGE–RAGE activation can enhance cytokine production and ROS accumulation, creating a microenvironment that favors TGF-β1 activation, extracellular matrix deposition, and fibrotic remodeling (145). Similarly, sympathetic overactivity can reinforce CSMC hypercontractility through norepinephrine-mediated α-adrenergic receptor activation, increasing intracellular calcium levels and opposing cavernous relaxation (146). These mechanisms converge on a common pathological endpoint: reduced smooth muscle relaxation, increased collagen deposition, decreased tissue distensibility, and impaired veno-occlusion (147).

In summary, CSMC abnormalities in varicocele-associated ED may involve apoptosis, phenotypic transformation, excessive contractility, and fibrosis. TNF-α-mediated caspase activation, PI3K/Akt inhibition, TGF-β1-driven extracellular matrix remodeling, RhoA/ROCK activation, AGE–RAGE inflammatory amplification, and sympathetic catecholamine signaling jointly contribute to the deterioration of corpus cavernosum structure and function. This vertically integrated interpretation avoids separating molecular pathways from their cellular consequences and highlights cavernous fibrosis and contractile imbalance as key pathological outcomes.

### 4.3 Inflammatory damage to penile nerves

Normal erectile function depends not only on endothelial relaxation and CSMC responsiveness but also on intact cavernous nerve signaling (111, 131). Penile nerves regulate neurotransmitter release, NO-mediated relaxation, and coordinated neurovascular coupling during erection (61, 84). Therefore, inflammatory or oxidative damage to cavernous nerves can contribute to neurogenic ED and further amplify vascular and smooth muscle dysfunction. Inflammatory cytokines may impair penile nerve integrity through several mechanisms. TNF-α and related pro-inflammatory mediators can inhibit nerve growth factor (NGF), which is essential for neuronal survival, axonal maintenance, and nerve regeneration (84). Reduced NGF signaling may weaken cavernous nerve repair capacity and increase vulnerability to inflammatory injury (84). In addition, inflammatory conditions can induce excessive inducible nitric oxide synthase (iNOS) activity, leading to high local NO production (148). Although NO is essential for normal erectile physiology, excessive or dysregulated NO production under inflammatory conditions may become neurotoxic and contribute to neuronal apoptosis (149).

Oxidative stress further aggravates neural injury (150). ROS generated during inflammatory activation can damage neuronal lipids, proteins, and mitochondrial structures, thereby impairing axonal conduction and neurotransmitter release (151, 152). Animal models of cavernous nerve injury have shown that oxidative stress markers increase in cavernous tissue and correlate with impaired erectile function (153, 154). Although some experimental studies have reported reduced cavernous nerve fiber density and impaired neurotransmitter release in varicocele-related settings, much of the current evidence remains derived from non-varicocele nerve injury models or broader ED research (111, 155). The molecular pathways discussed above may also contribute indirectly to neural injury. AGE–RAGE/NF-κB activation can maintain a chronic inflammatory environment that damages neural structures and impairs local repair responses (156). Sympathetic overactivity may further disturb neurovascular balance by favoring vasoconstrictive signaling and reducing the effectiveness of parasympathetic pro-erectile pathways (61, 146). Meanwhile, endothelial dysfunction and CSMC fibrosis can create a hostile microenvironment with reduced oxygen delivery and increased oxidative stress, further compromising nerve function (105). Thus, neural injury should be viewed as part of an integrated neurovascular disorder rather than an isolated anatomical event (84).

In summary, inflammatory neural injury in varicocele-associated ED may involve cytokine-mediated NGF suppression, iNOS-related neurotoxicity, oxidative neuronal damage, and impaired neurotransmitter release. These changes can disrupt neurovascular coupling and interact with endothelial dysfunction and CSMC remodeling to further impair erectile function. However, direct validation of penile neural injury in human varicocele-associated ED remains limited, and future studies should clarify whether neural impairment is a primary contributor or a secondary consequence of inflammatory vascular and structural damage.

### 4.4 Summary of the integrated testicular-penile pathological axis

The mechanisms described above suggest that varicocele may contribute to ED through a multi-level testicular–penile pathological axis centered on inflammation, oxidative stress, vascular dysfunction, smooth muscle remodeling, and neural injury (4, 24, 111). In this model, varicocele-induced venous congestion initiates local testicular hypoxia, hyperthermia, oxidative stress, immune-cell activation, and cytokine production (4, 43). These local events may then disseminate systemically through circulating inflammatory mediators and activated immune cells, which act as pathological messengers targeting the penile microenvironment (3, 43, 84).

**Figure 4:**
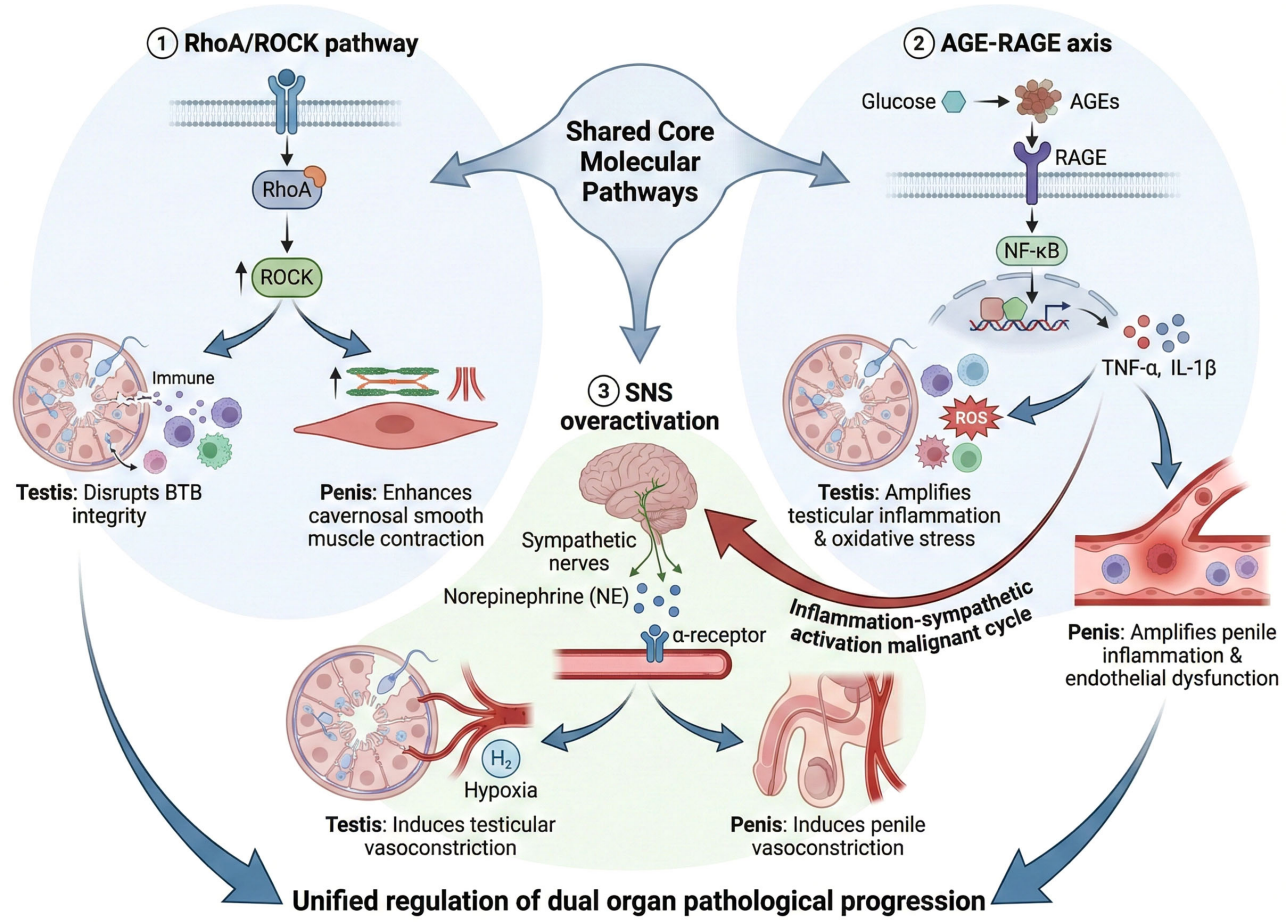
Schematic diagram of common molecular pathways linking testicular and penile pathologies in varicocele patients. Three pivotal molecular pathways mediate the pathological crosstalk between the testicular and penile microenvironments: (1) RhoA/ROCK pathway: In the testis, inflammatory activation of this pathway disrupts the blood-testis barrier (BTB) by altering Sertoli cell cytoskeletal dynamics; in the penis, it enhances cavernous smooth muscle contraction and suppresses eNOS activity, impairing vasodilation. (2) AGE-RAGE axis: Varicocele-induced oxidative stress promotes AGE formation, which binds to RAGE and activates the NF-κB pathway, perpetuating chronic inflammation and oxidative stress in both organs. (3) Sympathetic nervous system (SNS) overactivity: Elevated catecholamines (e.g., norepinephrine) induce vasoconstriction in testicular and penile vasculature, compromising testicular perfusion and penile erection. These shared pathways form a unified molecular basis for the comorbidity of spermatogenic impairment and erectile dysfunction in varicocele patients.

Within penile tissue, these systemic signals may induce three major pathological outcomes. First, endothelial dysfunction develops through TNF-α/IL-1β-mediated eNOS suppression, ROS-mediated NO depletion, ET-1 and angiotensin II activation, RhoA/ROCK signaling, AGE–RAGE/NF-κB amplification, and sympathetic catecholamine-driven vasoconstriction (3, 157–159). Second, CSMC abnormalities arise through TNF-α-induced apoptosis, PI3K/Akt inhibition, TGF-β1-driven phenotypic transformation, ROCK-mediated hypercontractility, and extracellular matrix deposition (160). Third, neural injury occurs through inflammatory suppression of NGF, iNOS-related neurotoxicity, oxidative stress, and impaired neurotransmitter release (61). Together, these processes lead to reduced arterial inflow, impaired cavernous relaxation, decreased tissue compliance, defective veno-occlusion, and ultimately ED (**Figure 4**).

This vertically integrated framework also clarifies the role of shared molecular pathways. RhoA/ROCK is not merely an independent pathway but a mediator of endothelial dysfunction, smooth muscle contraction, and fibrotic remodeling (**Figure 4**) (161). AGE–RAGE signaling functions as an amplifier of oxidative stress, NF-κB activation, and cytokine production, thereby affecting endothelial cells, smooth muscle cells, and possibly neural structures (162, 163). Sympathetic overactivity and catecholamines serve as hemodynamic and neuroimmune amplifiers that worsen vasoconstriction and reinforce inflammatory injury (146). By embedding these pathways into specific pathological outcomes, the narrative avoids repeating the same mechanisms across separate anatomical and molecular subsections (24).

It is important to define the evidence hierarchy clearly. The relationship between varicocele and spermatogenic impairment is strongly supported by clinical and experimental evidence, particularly through oxidative stress, inflammatory cytokines, blood-testis barrier disruption, and germ-cell injury. In contrast, the relationship between varicocele and ED is more tentative. Evidence for penile endothelial dysfunction, CSMC fibrosis, and neural injury in varicocele is derived predominantly from animal models, gene-knockout systems, and extrapolation from broader ED models, including diabetes, vascular injury, aging, and cavernous nerve injury. Therefore, the proposed testicular–penile axis should be regarded as biologically plausible and preclinically supported, but not yet definitively established in human populations. This graded interpretation distinguishes pathways supported by clinical and experimental evidence from those that remain primarily hypothetical or extrapolated from broader ED models (**Table 1**).

**Table 1:** Summary of proposed testicular-penile pathways and biomarkers.

Pathway/biomarker	Testicular evidence	Penile evidence	Study type	Level of evidence	Clinical relevance
Inflammatory Cytokines (TNF-α, IL-6)	Elevated in Varicocele Patients	Associated with ED (general literature)	Clinical + Experimental	Moderate	Potential Biomarker
Oxidative Stress (ROS)	Strong Evidence in Varicocele	Demonstrated in ED Models	Clinical + Animal	Moderate	Therapeutic Target
Blood-Testis Barrier Disruption	Clinical + Animal Studies	Hypothetical Systemic Effect	Animal + Indirect	Low	Mechanistic Hypothesis
Immune Cell Migration	Animal Models	Animal Studies	Animal	Low	Experimental
Endothelial Dysfunction	Limited Clinical Evidence	Well-Established in ED	Clinical + Extrapolated	Moderate	High Clinical Relevance
Smooth Muscle Remodeling	Experimental Evidence	ED Animal Models	Animal + In Vitro	Low-Moderate	Mechanistic
Neural Injury	Animal Studies	Experimental ED Models	Animal	Low	Hypothesis
RhoA/ROCK Pathway	Experimental Evidence	ED Models	Animal + In Vitro	Low-Moderate	Therapeutic Target
AGE-RAGE Pathway	Oxidative Stress Studies	ED Literature	Experimental	Low	Emerging
Sympathetic Overactivity	Limited Clinical Data	ED Literature	Mixed	Low	Hypothesis

## 5 Clinical evidence and biomarker research

Varicocele is a well-established contributor to male infertility and has also been associated with erectile dysfunction in some clinical and preclinical studies, with accumulating evidence suggesting a potential role for immune-inflammatory dysregulation in its pathophysiology (4, 11). Although some reports have described reduced erectile function scores, altered penile hemodynamic parameters, or improvement after varicocelectomy, the overall evidence base is heterogeneous and relies predominantly on observational or single-center studies. The investigation of clinical manifestations, inflammatory profiles, and potential biomarkers may facilitate deeper exploration of the mechanisms underlying varicocele-related reproductive impairments and provide insights for optimizing clinical diagnosis and prognosis (164). This section systematically summarizes the clinical evidence linking inflammatory markers to varicocele, the correlation between erectile function assessment and inflammatory parameters, as well as the latest advances in novel diagnostic and prognostic biomarkers, aiming to integrate current research findings and provide insights for future studies in this field.

### 5.1 Inflammatory marker profiles in semen and blood of patients with varicocele

The relationship between varicocele and male infertility has been extensively studied, particularly focusing on the inflammatory markers present in the semen and blood of affected individuals (3, 4). Multiple case-control studies have reported elevated levels of pro-inflammatory cytokines in patients with varicocele-associated infertility such as IL-6 and TNF-α in their seminal plasma (165–167). Additionally, ROS levels and leukocyte concentrations, primarily granulocytes, are markedly higher in these patients compared to fertile controls. Elevated inflammatory markers have been reported to correlate negatively with semen parameters, including sperm concentration, motility, and morphology, suggesting that increased inflammation may directly impair spermatogenesis and sperm function (22). In a cohort study, the oxidative stress index (OSI) and total oxidant status (TOS) were significantly elevated in patients with varicocele, highlighting a potential mechanism by which inflammation contributes to infertility (168). Furthermore, the presence of these inflammatory markers in seminal plasma serves not only as a diagnostic tool but also as a potential target for therapeutic interventions aimed at reducing oxidative stress and improving fertility outcomes (168).

In addition to local inflammatory responses in the semen, systemic inflammatory markers in the serum have also been linked to the severity of varicocele and the quality of semen. Studies have shown that serum levels of C-reactive protein (CRP) and IL-6 correlate with both the severity of varicocele and the decline in semen quality. For instance, a prospective cohort study indicated that patients with higher levels of CRP and IL-6 were more likely to experience significant reductions in semen quality, reinforcing the idea that systemic inflammation may exacerbate the local inflammatory environment within the reproductive tract (169). The neutrophil-lymphocyte ratio (NLR), a marker of systemic inflammation, has also been identified as a significant predictor of fertility outcomes post-varicocelectomy, suggesting that inflammatory status could influence surgical success rates (170).

Moreover, the interplay between oxidative stress and inflammation in patients with varicocele is further elucidated by the examination of free light chains (FLCs) of immunoglobulins, which serve as markers of low-grade inflammation. Studies have indicated that seminal levels of FLCs are elevated in varicocele patients compared to controls, and these levels correlate with total antioxidant capacity (TAC) and coenzyme Q10 (CoQ10) levels, both of which are crucial for sperm motility and overall reproductive health (171). The findings suggest that not only are inflammatory markers elevated in the context of varicocele, but they also interact with oxidative stress pathways, potentially leading to a compounded effect on male fertility.

In conclusion, the profiles of inflammatory markers in both semen and blood provide significant insights into the pathophysiology of infertility associated with varicocele. The consistent elevation of pro-inflammatory cytokines and oxidative stress indicators underscores the need for further research to elucidate the molecular mechanisms driving these changes and to explore potential therapeutic strategies aimed at mitigating inflammation and restoring fertility in affected patients. Understanding these interactions will be crucial for developing targeted interventions that can improve reproductive outcomes in men with varicocele. Taken together, inflammatory markers in semen and blood provide supportive clinical evidence for immune-inflammatory activation in varicocele, particularly in relation to spermatogenic impairment. However, their association with erectile dysfunction remains indirect and requires further validation.

### 5.2 Correlation between erectile function assessment and inflammatory parameters

The assessment of erectile function in patients with varicocele-related erectile dysfunction has garnered significant attention, particularly through the use of the International Index of Erectile Function (IIEF-5) questionnaire and color Doppler ultrasound. Studies have demonstrated that patients with erectile dysfunction associated with varicocele exhibit notably lower peak systolic velocity (PSV) in the penile cavernous arteries, indicating compromised arterial flow during the erection phase. Conversely, these patients often present with a higher resistance index (RI), suggesting increased vascular resistance and impaired blood flow dynamics, which are critical for achieving and maintaining an erection. This phenomenon underscores the intricate relationship between vascular health and erectile function, where inflammation plays a pivotal role. Preliminary research has indicated a negative correlation between serum levels of TNF-α and IIEF-5 scores, suggesting that elevated inflammatory markers may exacerbate erectile dysfunction. Specifically, higher TNF-α levels are associated with poorer erectile function, as reflected in lower IIEF-5 scores, while a positive correlation exists between TNF-α levels and RI, further supporting the hypothesis that inflammation mediates vascular dysfunction in these patients. This clinical evidence highlights the potential for targeting inflammatory pathways as a therapeutic strategy to improve erectile function in individuals suffering from varicocele-related erectile dysfunction. The interplay between inflammatory markers and erectile function necessitates further exploration into the underlying molecular mechanisms, as well as the development of targeted interventions aimed at mitigating inflammation to restore normal erectile function. Understanding this relationship not only enhances our comprehension of the pathophysiology of erectile dysfunction but also opens avenues for innovative treatment modalities that could significantly improve patient outcomes in this demographic.

### 5.3 Potential novel diagnostic and prognostic biomarkers

The exploration of novel biomarkers for assessing the immune-inflammatory status of the testicular-penile microenvironment in patients with varicocele is gaining traction, particularly in relation to ED and spermatogenic disorders. In addition to traditional inflammatory mediators, specific microRNAs (miRNAs) and proteins found in seminal plasma or serum are emerging as promising candidates for this purpose. For instance, miR-210, which is associated with hypoxic conditions, has been identified as a potential biomarker that reflects the hypoxic state of the testicular microenvironment, which is often exacerbated by varicocele. This miRNA’s dysregulation can lead to impaired spermatogenesis and contribute to the pathophysiology of ED (17). Furthermore, extracellular vesicles (EVs) are being investigated for their role in cell-cell communication and as carriers of bioactive molecules. These vesicles can encapsulate proteins, lipids, and nucleic acids, providing a snapshot of the cellular environment from which they originate. Studies have shown that the levels of specific proteins and nucleic acids within EVs vary significantly in men with varicocele, suggesting their potential utility as diagnostic biomarkers for male reproductive disorders (164).

Moreover, soluble receptors such as sRAGE (soluble receptor for advanced glycation end products) have been proposed as biomarkers that could indicate the degree of inflammation and oxidative stress within the testicular microenvironment. Elevated levels of sRAGE have been correlated with poor semen quality and may serve as a prognostic marker for treatment outcomes in varicocele patients (172). The integration of these biomarkers into clinical practice could allow for more personalized approaches to managing male infertility, particularly in cases where conventional semen analysis fails to reveal underlying pathophysiological mechanisms.

In addition to these molecular markers, advancements in proteomics and metabolomics are providing deeper insights into the biochemical milieu of the reproductive system. For example, proteomic analyses have identified differentially expressed proteins in the seminal plasma of varicocele patients, revealing alterations in pathways related to oxidative stress and mitochondrial dysfunction (173). Similarly, metabolomic profiling has uncovered specific metabolites that correlate with impaired sperm function, suggesting that these metabolic changes could serve as indicators of reproductive health (164).

The potential for AI in identifying novel biomarkers is also noteworthy. Recent studies have employed AI algorithms to analyze complex datasets, including clinical demographics and laboratory results, to uncover patterns and predict treatment responses in varicocele patients (172, 174, 175). This approach could revolutionize the way we assess and manage male infertility by enabling the identification of high-risk patients who may benefit from early intervention.

In conclusion, the identification of novel biomarkers such as specific miRNAs, proteins, and metabolites holds great promise for enhancing our understanding of the immune-inflammatory interactions within the testicular-penile microenvironment. These biomarkers not only have the potential to improve diagnostic accuracy but also to inform treatment decisions and prognostication in men suffering from varicocele-related reproductive issues. Future research should focus on validating these biomarkers in larger clinical cohorts and integrating them into routine clinical practice to optimize male fertility management. Overall, clinical and biomarker studies support the presence of immune-inflammatory dysregulation in varicocele, particularly in relation to spermatogenic impairment. However, the clinical evidence linking these biomarkers to erectile dysfunction remains limited and heterogeneous. Future large-scale prospective studies are required to validate these findings and establish clinically applicable biomarkers.

### 5.4 Critical appraisal of the clinical literature on varicocele and erectile dysfunction

A critical review of the available clinical literature indicates that the evidence supporting a direct relationship between varicocele and ED is suggestive but not conclusive. Most published studies are observational, frequently single-center, and often include limited sample sizes, which increases susceptibility to selection bias, residual confounding, and type I error (11, 176). In addition, study populations are commonly restricted to infertile men seeking specialist evaluation; accordingly, the reported prevalence and severity of ED may not reflect the broader population of men with varicocele (177–179).

Another major limitation is inadequate control for confounders. ED is a multifactorial disorder influenced by age, obesity, metabolic syndrome, cardiovascular risk, endocrine status, medication exposure, and psychosocial distress (180–182). Because male infertility itself may be accompanied by anxiety, depression, reduced self-esteem, and relationship stress, it is difficult to disentangle a direct organic effect of varicocele from infertility-related psychogenic or mixed etiologies of sexual dysfunction (183). Studies that do not systematically assess these variables may overestimate the independent contribution of varicocele.

Furthermore, clinical improvement after varicocelectomy should be interpreted cautiously. Although postoperative increases in testosterone levels and IIEF scores have been reported in some series, these studies are frequently uncontrolled or non-randomized, and the observed benefits may reflect regression to the mean, placebo effects, improved endocrine milieu, reduced pain burden, or psychological reassurance after treatment rather than direct reversal of a specific immune-inflammatory penile lesion. Thus, intervention studies provide supportive but not definitive evidence for causality.

Overall, the clinical literature currently supports three cautious conclusions. First, varicocele is strongly linked to impaired semen quality and testicular inflammatory-oxidative abnormalities. Second, an association between varicocele and ED has been reported in selected cohorts, but the magnitude and independence of this association remain uncertain. Third, the hypothesis that immune-inflammatory dysregulation forms a direct testis-penis pathological axis is biologically plausible and supported by preclinical data, yet still requires robust validation in well-designed human studies.

## 6 The impact of existing treatment strategies on immune-inflammatory interactions

Growing evidence indicates that immune-inflammatory dysregulation plays a pivotal role in the pathogenesis of varicocele-related complications, including male infertility and erectile dysfunction (4, 27). Existing treatment strategies for varicocele are not only designed to correct the underlying pathological abnormalities but also to modulate the intricate interplay between the immune system and inflammatory responses, thereby exerting comprehensive therapeutic effects (4, 7, 184). In the following sections, we will elaborate on the specific impacts of two major therapeutic approaches—varicocele ligation surgery and adjuvant therapy with antioxidants or anti-inflammatory agents—on immune-inflammatory interactions, as well as their potential molecular mechanisms and clinical implications.

### 6.1 Systemic anti-inflammatory effects of varicocele ligation surgery

Varicocele, a condition characterized by the enlargement of veins within the scrotum, has been increasingly recognized as a significant contributor to male infertility. Surgical intervention, particularly high ligation of the spermatic vein, has been shown to yield substantial benefits, not only in local venous drainage but also in systemic inflammatory responses. Successful varicocele ligation has been associated with a marked decrease in the levels of pro-inflammatory cytokines such as IL-6 and TNF-α in both serum and seminal plasma following the procedure. These findings suggest that the surgical correction of varicocele alleviates the localized inflammatory environment within the testicular microenvironment, which is often exacerbated by oxidative stress and inflammatory mediators. The reduction of ROS levels post-surgery further supports the notion that varicocele ligation can lead to a significant improvement in both local and systemic inflammation, contributing to enhanced testicular function and overall reproductive health (81).

Moreover, the attenuation of inflammation in the testicular microenvironment may have far-reaching implications for erectile function, as evidenced by improvements in erectile function parameters, such as the International Index of Erectile Function (IIEF) scores, observed in some studies post-surgery. This suggests that the benefits of varicocele ligation extend beyond the immediate anatomical corrections, positively influencing the distal penile microenvironment as well. The interplay between local testicular inflammation and systemic inflammatory states is complex, but it is clear that the surgical intervention has the potential to restore a more favorable immune milieu, which is crucial for both spermatogenesis and erectile function. The systemic anti-inflammatory effects observed after varicocele ligation may be attributed to a reduction in the production of anti-sperm antibodies (ASAs) and pro-inflammatory cytokines that are often elevated in patients with varicocele, thereby restoring the delicate balance necessary for optimal reproductive function (3).

In summary, the systemic anti-inflammatory effects of varicocele ligation surgery underscore the importance of addressing this condition not only to improve fertility outcomes but also to enhance overall male reproductive health. The reduction in inflammatory markers and ROS levels post-surgery indicates a restoration of testicular function, which can lead to improvements in erectile function and a reduction in the risk of infertility associated with varicocele. Future studies should focus on elucidating the precise molecular mechanisms underlying these systemic effects, as well as the long-term benefits of surgical intervention on male reproductive health. Understanding these pathways will be essential for developing targeted therapies aimed at mitigating the adverse effects of varicocele and improving outcomes for affected individuals (185).

### 6.2 Adjuvant therapy with antioxidants and anti-inflammatory drugs

The use of adjuvant therapies, particularly antioxidants and anti-inflammatory drugs, has gained traction in the management of conditions associated with varicocele, such as male infertility and erectile dysfunction. Commonly utilized medications, including L-carnitine, theobromine, and non-steroidal anti-inflammatory drugs (NSAIDs), have demonstrated mechanisms that involve the inhibition of oxidative stress and inflammatory responses. For instance, L-carnitine has been shown to enhance mitochondrial function and improve sperm quality by reducing ROS levels, thereby mitigating oxidative stress that can adversely affect spermatogenesis (81). Similarly, theobromine exhibits anti-inflammatory properties that may contribute to improved testicular function by reducing inflammation in the microenvironment of the testes (186). NSAIDs, while primarily used for pain relief, also play a role in decreasing local inflammation and may indirectly support erectile function by improving blood flow and reducing tissue damage associated with chronic inflammatory states (187).

The theoretical benefits of these agents extend beyond the improvement of semen parameters; they may also alleviate systemic inflammatory burdens that can impact erectile function. The interplay between oxidative stress, inflammation, and erectile dysfunction is complex, and while studies suggest that reducing oxidative stress can enhance erectile function, definitive clinical evidence is still required to substantiate these claims (188). For example, studies on resveratrol have indicated its potential to improve endothelial function and reduce inflammatory markers in the urogenital tract, which could translate into better erectile function (188). However, the bioavailability of such compounds often limits their effectiveness, necessitating further research to optimize dosing regimens and delivery methods to achieve clinically significant outcomes.

Moreover, the combination of these agents with lifestyle modifications, such as exercise and dietary changes, may enhance their efficacy. Exercise has been shown to exert beneficial effects on oxidative stress and inflammation, promoting overall health and potentially improving both spermatogenic and erectile functions (189). Therefore, while the use of antioxidants and anti-inflammatory drugs holds promise as an adjunctive therapy in managing varicocele-related conditions, more targeted clinical studies are essential to confirm their effectiveness and to elucidate the underlying molecular mechanisms involved. The integration of these therapies into standard treatment protocols could provide a multifaceted approach to addressing the complications associated with varicocele, ultimately leading to improved reproductive and sexual health outcomes for affected individuals.

## 7 Potential new therapeutic strategies targeting immune-inflammatory interactions

The intricate crosstalk between immune responses and inflammatory processes plays a pivotal role in the pathophysiological progression of varicocele and its associated complications, including testicular dysfunction, spermatogenic impairment, and erectile dysfunction (4, 12, 99). Accumulating preclinical and clinical evidence has underscored that dysregulated immune-inflammatory networks are not merely secondary manifestations but core drivers of tissue damage and functional deterioration in the testicular-penile microenvironment (3, 74). Consequently, targeting the key molecular nodes and cellular mediators of these interactive pathways has emerged as a promising direction for the development of novel, effective therapeutic interventions (7, 74, 84). Below, we summarize and discuss the latest advances in three major categories of potential therapeutic strategies, including specific inhibitors of inflammatory pathways, stem cell-based immunomodulatory therapy, and epigenetic regulation interventions, with a focus on their mechanisms of action, preclinical efficacy, and translational potential in the management of varicocele-related disorders.

### 7.1 Specific inhibitors of inflammatory pathways

The exploration of specific inflammatory pathway inhibitors, particularly monoclonal antibodies or receptor antagonists targeting key inflammatory mediators such as TNF-α and IL-1β, has gained traction in the context of various diseases, including rheumatoid arthritis. These agents have shown promise in modulating immune responses and reducing inflammatory cascades. In the context of varicocele-related testicular-penile comorbid models, the efficacy of these inhibitors is currently under investigation. The rationale behind this approach lies in the understanding that chronic inflammation plays a pivotal role in the pathophysiology of varicocele, leading to oxidative stress and subsequent impairment of spermatogenesis and erectile function. By selectively inhibiting these inflammatory mediators, it is hypothesized that one could restore normal function in the testicular-penile microenvironment, potentially alleviating associated conditions such as erectile dysfunction and spermatogenic failure. Research is ongoing to evaluate the therapeutic potential of these agents in preclinical models, which may eventually translate into clinical applications. The ability to modulate specific inflammatory pathways could lead to more targeted and effective treatments for patients suffering from varicocele and its complications, providing a promising avenue for future therapeutic strategies.

In addition to monoclonal antibodies, small molecule inhibitors such as Rho-associated protein kinase (ROCK) inhibitors, including fasudil, have emerged as potential therapeutic agents. Animal studies have demonstrated that ROCK inhibitors can simultaneously protect testicular spermatogenic function and improve erectile function by enhancing cavernous smooth muscle relaxation. The mechanism of action is believed to involve the modulation of vascular tone and the enhancement of blood flow to the erectile tissues, thereby improving erectile function. Furthermore, ROCK inhibitors may exert protective effects on the germ cells within the testes, potentially reversing the negative impact of varicocele-induced oxidative stress. The dual action of these small molecules not only addresses the erectile dysfunction associated with varicocele but also targets the underlying spermatogenic impairment. This dual therapeutic approach highlights the potential for ROCK inhibitors to serve as a cornerstone in the management of varicocele-related complications, paving the way for future clinical trials aimed at establishing their efficacy and safety in human populations. As research progresses, the integration of these specific inflammatory pathway inhibitors into treatment protocols for varicocele may hold the key to improving reproductive health outcomes and overall quality of life for affected individuals.

### 7.2 The immunomodulatory role of stem cell therapy

Mesenchymal stem cells (MSCs) have garnered significant attention in recent years due to their potent anti-inflammatory and immunoregulatory properties. These cells can modulate immune responses through the secretion of paracrine factors such as TSG-6 and PGE2, which are known to inhibit macrophage activation and T cell proliferation. The immunomodulatory effects of MSCs are primarily mediated by their ability to interact with various immune cells, influencing their behavior and the local microenvironment. For instance, MSCs can promote the polarization of macrophages towards an anti-inflammatory M2 phenotype while simultaneously suppressing the pro-inflammatory M1 phenotype, thereby reducing the overall inflammatory response. This dual action not only mitigates inflammation but also fosters a more conducive environment for tissue repair and regeneration, making MSCs a promising therapeutic option for various inflammatory conditions, including those associated with varicocele-induced testicular dysfunction (190).

Animal studies have further elucidated the therapeutic potential of MSCs in the context of varicocele. Research indicates that both systemic and local administration of MSCs can significantly reduce testicular inflammation and oxidative damage in varicocele models, as evidenced by improved histological outcomes and reduced markers of oxidative stress. For example, in a rat model of varicocele, MSC treatment led to a notable decrease in inflammatory cytokines and an increase in antioxidant levels, suggesting that MSCs can effectively counteract the oxidative stress associated with varicocele-induced testicular injury. Moreover, the administration of MSCs not only alleviates inflammation but also addresses fibrotic changes in the corpus cavernosum, which is crucial for restoring erectile function. This “two birds with one stone” approach highlights the multifaceted benefits of MSC therapy in addressing both inflammatory and fibrotic components within the testicular microenvironment (191).

The therapeutic efficacy of MSCs is further enhanced by their ability to secrete EVs that carry bioactive molecules, including cytokines and growth factors, which contribute to their immunomodulatory effects. Recent findings suggest that MSC-derived EVs can ameliorate inflammation and promote tissue repair by modulating the activity of immune cells and enhancing angiogenesis. In the context of varicocele, MSC-EVs have been shown to reduce inflammation and oxidative stress in testicular tissues, thereby supporting spermatogenesis and improving overall testicular function. This underscores the potential of MSC-derived EVs as a cell-free therapeutic strategy, which may circumvent some of the challenges associated with direct MSC administration, such as cell survival and engraftment issues.

In summary, the immunomodulatory role of MSC therapy presents a promising avenue for the treatment of varicocele-related disorders. By leveraging their anti-inflammatory properties and ability to promote tissue regeneration, MSCs offer a multifaceted approach to restoring testicular function and addressing the underlying pathophysiological changes associated with varicocele. Continued research into the mechanisms of action and optimal delivery methods for MSCs and their derivatives will be crucial in translating these findings into clinical practice, ultimately improving outcomes for patients suffering from varicocele-induced infertility and erectile dysfunction (192).

### 7.3 Epigenetic regulation interventions

The role of epigenetic factors, particularly miRNAs, in the regulation of inflammatory networks within the testicular-penile microenvironment of varicocele patients has garnered increasing attention in recent years. Given the significant impact of miRNAs on gene expression and their involvement in various biological processes, the development of miRNA-based agonists or antagonists, such as antagomir-155, represents a promising novel direction for precisely modulating the immune-inflammatory interactions within this microenvironment. Varicocele, characterized by the abnormal dilation of the pampiniform plexus, has been linked to male infertility through mechanisms that include oxidative stress, inflammation, and impaired spermatogenesis (193). The dysregulation of miRNAs can exacerbate these conditions by influencing the expression of genes involved in inflammation and immune responses. For instance, miR-34a has been implicated in the apoptotic pathways within testicular cells, with elevated levels correlating with increased oxidative stress and cellular damage in varicocele (194). By targeting specific miRNAs, researchers aim to restore balance to the inflammatory processes that contribute to the pathophysiology of conditions like varicocele and its associated complications, including erectile dysfunction and spermatogenic failure.

The potential for miRNA-based therapies lies in their ability to fine-tune the immune response, promoting a more favorable environment for spermatogenesis and erectile function. For example, the application of antagomirs, which are designed to inhibit specific miRNAs, could reduce the expression of pro-inflammatory cytokines and restore normal testicular function (195). Furthermore, the interplay between miRNAs and epigenetic modifications, such as DNA methylation and histone modifications, adds another layer of complexity to the regulation of inflammation in the testicular microenvironment (196). Understanding these interactions could lead to the identification of biomarkers for assessing the severity of varicocele and its impact on fertility, as well as guide the development of targeted epigenetic therapies.

Moreover, the therapeutic implications of miRNA modulation extend beyond varicocele. The emerging field of nutri-epigenomics highlights how dietary components can influence the expression of miRNAs and other epigenetic factors, potentially mitigating the effects of oxidative stress and inflammation associated with male infertility (197). This suggests that lifestyle interventions, combined with targeted miRNA therapies, could offer a comprehensive approach to managing conditions like varicocele and its sequelae. Future research should focus on elucidating the specific roles of various miRNAs in the context of testicular inflammation and spermatogenesis, as well as exploring the feasibility of integrating dietary strategies with miRNA-based therapies to enhance reproductive health outcomes in affected individuals.

In conclusion, the exploration of epigenetic regulation through miRNA interventions presents a promising avenue for addressing the immune-inflammatory interactions within the testicular-penile microenvironment of varicocele patients. By harnessing the power of miRNAs, it may be possible to develop precise therapeutic strategies that not only alleviate the inflammatory processes contributing to male infertility but also promote overall reproductive health. Continued research in this area is essential to validate these approaches and translate them into clinical practice, ultimately improving the quality of life for men suffering from varicocele and its associated complications.

## 8 Future research directions and challenges

Despite significant advances in elucidating the role of immune-inflammatory dysregulation in varicocele and its associated reproductive complications, including spermatogenic impairment and erectile dysfunction, several critical knowledge gaps and clinical challenges remain to be addressed (3, 4). These unresolved issues hinder the translation of basic research findings into effective clinical practice and personalized therapeutic strategies. To move the field forward, future investigations should focus on integrating cutting-edge technological approaches, strengthening clinical evidence, and optimizing comprehensive management paradigms that target the underlying pathophysiological mechanisms. The following sections outline the key research directions and challenges that need to be prioritized to advance our understanding and improve patient care in this field.

### 8.1 Multi-omics integration and systems biology analysis

The integration of multi-omics approaches, including transcriptomics, proteomics, and metabolomics, represents a crucial advancement in understanding the immune-inflammatory interactions within the testicular-penile microenvironment of patients with varicocele. Future research should prioritize comprehensive analyses of various biological samples from the same patient, such as testicular tissue, seminal plasma, blood, and, where feasible, penile cavernous tissue. This holistic strategy aims to construct a panoramic molecular map that elucidates the intricate interplay of immune and inflammatory responses. By employing high-throughput sequencing and mass spectrometry techniques, researchers can identify and quantify the expression of genes, proteins, and metabolites that are pivotal in the pathophysiology of erectile dysfunction and spermatogenic impairment associated with varicocele. For instance, recent studies have highlighted the role of specific proteins linked to oxidative stress and inflammation, such as stathmin and selenoprotein P, which are differentially expressed in varicocele patients before and after treatment (198). The identification of these core regulatory nodes is essential for understanding how varicocele affects male reproductive health and can lead to the development of targeted therapeutic strategies. Moreover, the application of systems biology frameworks allows for the integration of diverse datasets, enabling the identification of potential biomarkers and therapeutic targets that can be further validated in clinical settings. This integrated approach not only enhances our understanding of the molecular mechanisms underlying varicocele-related reproductive dysfunction but also facilitates the development of personalized medicine strategies that cater to the unique biological profiles of individual patients. Ultimately, the combination of multi-omics data with advanced bioinformatics tools will provide deeper insights into the complex biological networks involved in varicocele, paving the way for innovative interventions that could significantly improve patient outcomes.

### 8.2 Establishing more comprehensive clinical correlation studies

The establishment of comprehensive clinical correlation studies is essential for advancing our understanding of the interactions between inflammation and clinical outcomes in patients with varicocele. To achieve this, large-scale, prospective cohort studies should be initiated, focusing on long-term follow-up of varicocele patients. These studies would involve dynamic monitoring of various parameters, including inflammatory biomarkers, semen quality, hormonal levels, and erectile function over time. By employing a longitudinal approach, researchers can elucidate the causal relationships and temporal dynamics between inflammatory processes and clinical outcomes, such as fertility and erectile dysfunction. For instance, previous studies have indicated that varicocele is associated with increased levels of inflammatory markers, which may correlate with impaired spermatogenesis and erectile function (199). However, the precise mechanisms through which these inflammatory responses affect clinical outcomes remain poorly understood. Therefore, it is crucial to systematically collect data on inflammatory markers, such as cytokines and chemokines, alongside clinical assessments of semen parameters and erectile function. This integrative approach could provide valuable insights into the pathophysiology of varicocele-related complications and help identify potential therapeutic targets. Furthermore, the incorporation of advanced imaging techniques, such as dynamic contrast-enhanced MRI, could enhance our understanding of testicular perfusion and its relationship with inflammatory states in varicocele patients (200). Ultimately, these comprehensive studies should aim to establish robust predictive models that can guide clinical decision-making and improve the management of varicocele and its associated complications.

### 8.3 Development of comprehensive management strategies for comorbidities

The intricate relationship between immune-inflammatory interactions and conditions such as infertility and erectile dysfunction necessitates a shift in clinical management paradigms. Future clinical strategies should not isolate these conditions but rather adopt a comprehensive approach that addresses the underlying mechanisms linking them. Understanding the immune-inflammatory interactions in the testicular-penile microenvironment is pivotal. For instance, varicocele, a common condition affecting male fertility, is associated with increased oxidative stress and inflammation, which can adversely affect spermatogenesis and erectile function (53). This suggests that management should include not only surgical interventions, such as varicocelectomy, but also the adjunctive use of targeted anti-inflammatory and antioxidant therapies. Such combined strategies could mitigate the inflammatory milieu and improve both spermatogenic and erectile functions.

Moreover, lifestyle modifications play a crucial role in managing these comorbidities. Interventions aimed at reducing systemic inflammation, such as weight loss, dietary changes, and increased physical activity, could significantly enhance reproductive health outcomes. For instance, weight reduction has been shown to lower inflammatory markers and improve hormonal profiles, which are beneficial for both fertility and erectile function (201). The integration of lifestyle interventions into treatment plans could thus serve as a foundational aspect of comprehensive management strategies.

Additionally, the development of personalized treatment plans that consider individual patient profiles, including genetic predispositions and specific inflammatory markers, can enhance the effectiveness of interventions. Recent studies have highlighted the importance of metabolic profiling in understanding the pathophysiological mechanisms underlying varicocele-associated infertility (202). By identifying specific metabolic derangements, clinicians can tailor interventions that address these abnormalities, potentially restoring normal sperm function and improving erectile capabilities.

Furthermore, multidisciplinary collaboration among urologists, endocrinologists, nutritionists, and mental health professionals is essential in managing patients with these complex conditions. Such collaboration can facilitate a holistic approach to treatment, ensuring that all aspects of a patient’s health are considered. For example, addressing psychological factors such as stress and anxiety, which are often exacerbated by fertility issues and erectile dysfunction, can improve overall treatment outcomes (203).

In conclusion, the development of comprehensive management strategies for comorbidities associated with immune-inflammatory interactions should focus on an integrated approach that combines surgical, pharmacological, and lifestyle interventions. By understanding the complex interplay of these factors, healthcare providers can better protect and improve testicular and penile functions, ultimately enhancing the quality of life for affected individuals. Future research should continue to explore these interactions and refine management strategies to optimize patient outcomes in the context of male reproductive health.

## 9 Discussion

**Figure 5:**
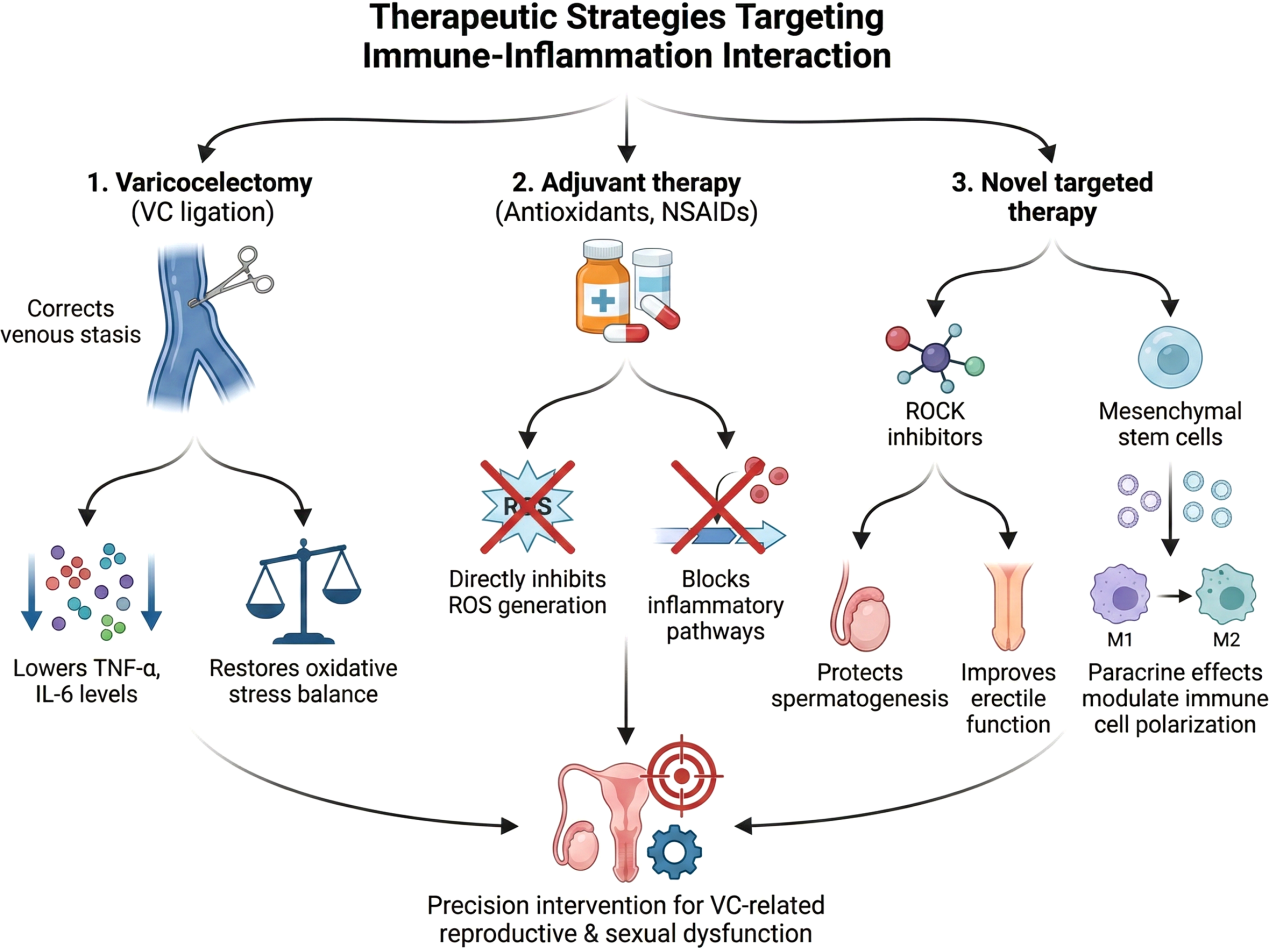
Schematic diagram of the integrated immune-inflammatory hypothesis of the testicular-penile pathological axis in varicocele patients. Varicocele-induced venous congestion in the testis triggers a cascade of immune-inflammatory and oxidative stress responses: (1) Local effects: Elevated temperature, hypoxia, and metabolic waste accumulation activate immune cells, induce pro-inflammatory cytokine (TNF-α, IL-1β, IL-6) release and ROS overproduction, disrupting the blood-testis barrier (BTB) and impairing spermatogenesis; (2) Systemic dissemination: Testicular-derived inflammatory mediators and activated immune cells enter the circulation, acting as “pathological messengers” to target the penile microenvironment; (3) Penile local damage: Systemic inflammatory signals induce endothelial dysfunction (reduced eNOS/NO), cavernous smooth muscle cell apoptosis/fibrosis, and penile nerve injury, leading to erectile dysfunction; (4) Common molecular pathways (RhoA/ROCK, AGE-RAGE, sympathetic overactivity) amplify damage in both organs. Collectively, these processes form a “testicular-penile pathological axis” centered on chronic inflammation and oxidative stress, explaining the comorbidity of spermatogenic impairment and erectile dysfunction in varicocele patients.

This review systematically elucidates the biologically plausible immune-inflammatory interactions within the testicular-penile microenvironment in varicocele patients and deciphers the mechanistically supported core molecular pathways underlying spermatogenic impairment and the proposed association with ED. The existing body of evidence supports the concept that varicocele is not merely a localized vascular abnormality of the pampiniform plexus but a condition associated with immune-inflammatory dysregulation, which may exert cascading detrimental effects on both testicular reproductive function and penile erectile physiology based primarily on preclinical data (4, 12, 99). The central thesis of this review—that chronic inflammation and oxidative stress act as plausible mechanistic links connecting testicular and penile pathologies—complements and extends the traditional understanding of varicocele as an isolated testicular disorder, and provides a novel integrative synthesis for exploring the comprehensive impact of varicocele on male reproductive health (**Figure 5**).

A pivotal finding highlighted in this review is the initiation of a multi-faceted pathological cascade in the testicular microenvironment by varicocele-induced venous congestion: elevated local temperature, hypoxia, and metabolic waste accumulation trigger immune cell infiltration and activation, leading to the overproduction of pro-inflammatory cytokines (TNF-α, IL-1β, IL-6) and ROS. This process disrupts the redox balance, impairs the structural and functional integrity of the blood-testis barrier (BTB), and induces autoimmune responses, collectively compromising the survival and maturation of germ cells and the secretory function of Sertoli and Leydig cells (12). What is particularly noteworthy is that these testicular-derived inflammatory mediators and activated immune cells may, in preclinical models, enter the systemic circulation, serving as “pathological messengers” to propagate inflammatory signals to the penile microenvironment (4, 204). This hypothesized systemic dissemination of inflammation constitutes the mechanistically plausible pathophysiological bridge between testicular dysfunction and penile ED, a mechanism that has long been underrecognized in traditional research focusing solely on local testicular changes (4, 204). However, direct clinical confirmation of this “testis-penis pathological axis” in humans remains incomplete, and clinical studies supporting a strong independent varicocele–ED association are limited and heterogeneous.

In the penile microenvironment, preclinically demonstrated systemic inflammatory signals induce a series of structural and functional abnormalities that may lead to ED, including endothelial cell dysfunction, phenotypic transformation and apoptosis of cavernous smooth muscle cells (CSMCs), and inflammatory damage to penile nerves (61, 204). The downregulation of endothelial nitric oxide synthase (eNOS) activity and reduced nitric oxide (NO) bioavailability—caused by pro-inflammatory cytokines—are the core preclinical vascular mechanisms of ED in varicocele patients, as NO is the key mediator of penile vasodilation and erection maintenance (204). Concurrently, TNF-α and TGF-β1 drive CSMC apoptosis and fibroblast-like phenotypic transformation, resulting in cavernous fibrosis and reduced tissue distensibility, while inflammatory cytokines and excessive ROS impair penile nerve integrity and neurotransmitter release, exacerbating neurogenic ED in animal studies (205). These pathological changes in the penis are hypothesized to be distal manifestation of the systemic immune-inflammatory network activated by varicocele, forming a conceptual “testis-penis pathological axis” centered on inflammation and oxidative stress (205).

The identification of preclinically validated common molecular pathways linking testicular and penile pathologies—including the RhoA/ROCK pathway, the AGE-RAGE axis, and sympathetic nervous system (SNS) overactivity—further clarifies the biologically unified molecular basis of varicocele-related reproductive dysfunction (128, 206, 207). The RhoA/ROCK pathway, for instance, mediates BTB disruption in the testis and enhanced cavernous smooth muscle contraction in the penis through a conserved inflammatory activation mechanism, making it a promising dual-target for the treatment of both spermatogenic impairment and ED in preclinical models (208). The activation of the AGE-RAGE axis amplifies chronic inflammation and oxidative stress in both microenvironments, while SNS overactivity and elevated catecholamines induce vasoconstriction in both testicular and penile vasculature, forming a vicious cycle of sympathetic activation and inflammatory damage (207). These shared molecular pathways provide a mechanistic rationale for the clinical co-occurrence of infertility and ED in some varicocele patients but do not confirm a direct causal relationship in humans.

Clinical evidence and biomarker research further validate the clinical significance of immune-inflammatory dysregulation in varicocele. The evidence linking these markers to ED in varicocele patients remains preliminary. The consistent elevation of pro-inflammatory cytokines (TNF-α, IL-6) and oxidative stress markers in the semen and serum of varicocele patients correlates negatively with semen quality and weakly or inconsistently with IIEF-5 scores, while novel biomarkers such as miR-210, sRAGE, and extracellular vesicles (EVs) exhibit preclinical promise for the early diagnosis and prognostic evaluation of varicocele-related complications (4, 209). Animal models, especially gene knockout and transgenic models, have played an indispensable role in mechanistic verification, confirming the causal relationship between key inflammatory mediators (e.g., TNF-α) and testicular-penile damage in preclinical settings, and providing a preclinical platform for evaluating the efficacy of potential therapeutic agents (7, 210). Existing treatment strategies, including varicocele ligation and adjuvant antioxidant/anti-inflammatory therapy, exert therapeutic effects primarily by inhibiting systemic inflammation and restoring redox balance, with clinical data demonstrating their ability to reduce inflammatory marker levels and improve both semen parameters and modestly or inconsistently improve erectile function (4, 54, 211). However, the current clinical efficacy of these treatments remains heterogeneous, and there is a lack of personalized treatment protocols based on immune-inflammatory status, highlighting the need for further optimization of existing therapies.

The emerging potential therapeutic strategies targeting immune-inflammatory interactions—including specific inflammatory pathway inhibitors, stem cell-based immunomodulatory therapy, and epigenetic regulation—represent the future development direction of varicocele treatment (212, 213). ROCK inhibitors, as small molecule inhibitors with dual effects on the testis and penis, have shown promising preclinical efficacy in protecting spermatogenic function and improving erectile function (214, 215). Mesenchymal stem cell (MSC) therapy exerts anti-inflammatory and tissue repair effects through paracrine factors and EVs, providing a novel cell-based therapy for the treatment of severe varicocele-related damage (216). Epigenetic regulation, particularly miRNA-based interventions (e.g., antagomir-155), enables precise modulation of the immune-inflammatory network, offering a personalized therapeutic approach for varicocele patients with specific epigenetic dysregulation (217–219). These novel strategies break through the limitations of traditional symptomatic treatment and focus on targeting the core pathogenic mechanism of immune-inflammatory dysregulation, holding great translational potential for improving the clinical outcomes of varicocele patients.

Despite the significant advances in understanding the immune-inflammatory mechanisms of varicocele summarized in this review, several critical knowledge gaps and challenges remain to be addressed. First, the precise molecular regulatory network of the hypothetical “testis-penis pathological axis” is not yet fully elucidated, and the specific signal transduction pathways by which testicular inflammatory mediators regulate penile microenvironmental changes require further in-depth exploration using multi-omics and systems biology approaches in clinical cohorts. Second, current clinical biomarker research is mostly in the exploratory stage, and large-scale prospective cohort studies are needed to validate the diagnostic and prognostic value of novel biomarkers and establish standardized detection methods. Third, the development of novel targeted therapies is still in the preclinical stage, and there is an urgent need for well-designed clinical trials to evaluate their safety and efficacy in humans. Fourth, the clinical management of varicocele currently lacks a comprehensive assessment system integrating immune-inflammatory status, and the development of personalized treatment protocols based on individual inflammatory profiles remains a major clinical challenge. Additionally, the long-term systemic impact of varicocele-induced immune-inflammatory dysregulation on male overall health (e.g., cardiovascular disease risk) remains unclear and warrants further epidemiological and mechanistic research.

Despite the emerging insights summarized in this review, several limitations should be acknowledged. First, the available clinical studies investigating the relationship between varicocele and erectile dysfunction remain heterogeneous in terms of study design, patient characteristics, diagnostic criteria, and outcome assessment. Such heterogeneity limits the comparability of findings and reduces the strength of clinical conclusions. In particular, variations in varicocele grading, patient age, comorbidities, and assessment tools for erectile function contribute to inconsistencies across studies. Second, the number of prospective and longitudinal clinical studies directly evaluating the interaction between testicular pathology and penile dysfunction in varicocele patients remains limited. Most currently available data are derived from cross-sectional or observational studies, which preclude definitive causal inference. In addition, interventional studies assessing changes in erectile function following varicocele treatment are still relatively scarce and often involve small sample sizes. Third, many of the proposed molecular mechanisms described in this review are primarily derived from animal models, *in vitro* experiments, or extrapolation from broader erectile dysfunction research. Although these findings provide biologically plausible explanations, their direct applicability to human varicocele-associated erectile dysfunction remains uncertain. This highlights an important translational gap between mechanistic findings and clinical application. Furthermore, emerging biomarkers and therapeutic targets discussed in this review remain largely exploratory. Their clinical utility has not yet been validated in large-scale studies, and standardized diagnostic or therapeutic strategies have not been established. Future research should therefore focus on well-designed prospective clinical studies, standardized patient selection criteria, and translational investigations integrating clinical and experimental data. Such efforts will be essential to validate the proposed testicular–penile pathological axis and to clarify its clinical significance.

In conclusion, immune-inflammatory interactions within the testicular-penile microenvironment are biologically plausible core pathophysiological mechanisms underlying varicocele-related spermatogenic impairment and the proposed association with erectile dysfunction, and the hypothesized systemic dissemination of inflammation forms a mechanistically supported pathological link between the two organs. The identification of common molecular pathways and novel biomarkers provides a theoretical basis for the early diagnosis and targeted treatment of varicocele, while emerging therapeutic strategies targeting immune-inflammatory dysregulation offer promising avenues for improving the comprehensive clinical outcomes of varicocele patients. Future research should focus on integrating multi-omics technologies to decipher the precise molecular regulatory network of the testicular-penile axis, validating novel biomarkers in large clinical cohorts, accelerating the translational research of novel targeted therapies, and establishing a comprehensive personalized management system based on immune-inflammatory status. By addressing these challenges, we can further advance the understanding and treatment of varicocele, and ultimately improve the reproductive health and quality of life of male patients with varicocele.

## 10 Literature search strategy

This narrative review was based on a literature search conducted in the PubMed database covering studies published from 1999 to 2026. The search terms included “Varicocele”, “Immune-inflammatory interaction”, “Testicular microenvironment”, “Penile microenvironment”, “Erectile dysfunction”, “Spermatogenic impairment”, and “Molecular mechanisms”. Relevant clinical studies, experimental studies, and review articles were screened and selected based on their relevance to the topic.
